# COUPY Coumarins as Novel Mitochondria-Targeted Photodynamic Therapy Anticancer Agents

**DOI:** 10.1021/acs.jmedchem.1c01254

**Published:** 2021-11-19

**Authors:** Enrique Ortega-Forte, Anna Rovira, Albert Gandioso, Joaquín Bonelli, Manel Bosch, José Ruiz, Vicente Marchán

**Affiliations:** † Departamento de Química Inorgánica, Universidad de Murcia and Institute for Bio-Health Research of Murcia (IMIB-Arrixaca), Campus de Espinardo, Murcia E-30071, Spain; ‡ Departament de Química Inorgànica i Orgànica, Secció de Química Orgànica, IBUB, Universitat de Barcelona, Martí i Franqués 1−11, Barcelona E-08028, Spain; § Unitat de Microscòpia Òptica Avançada, Centres Científics i Tecnològics, Universitat de Barcelona, Av. Diagonal 643, Barcelona E-08028, Spain

## Abstract

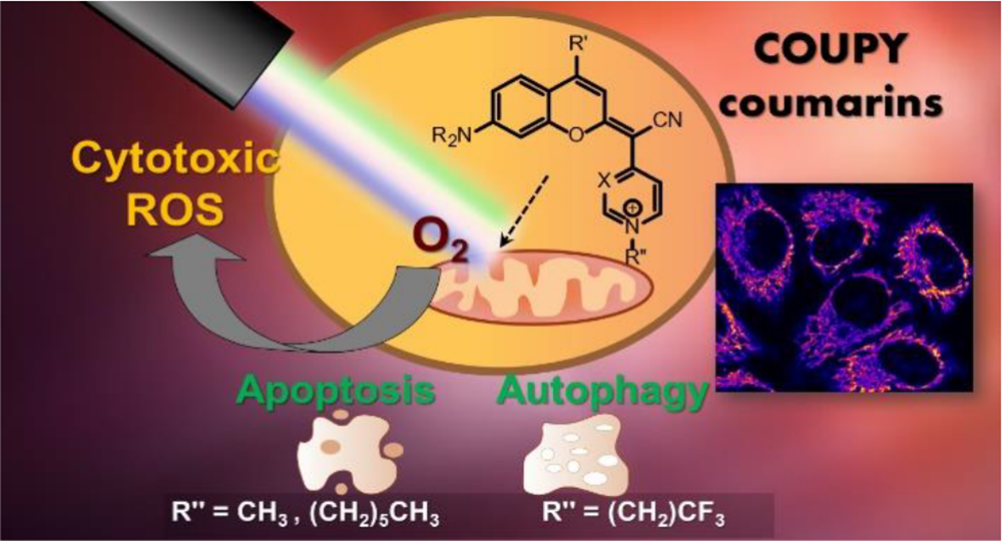

Photodynamic therapy (PDT) for cancer treatment has drawn increased attention over the last decades. Herein, we introduce a novel family of low-molecular-weight coumarins as potential PDT anticancer tools. Through a systematic study with a library of 15 compounds, we have established a detailed structure–activity relationship rationale, which allowed the selection of three lead compounds exhibiting effective in vitro anticancer activities upon visible-light irradiation in both normoxia and hypoxia (phototherapeutic indexes up to 71) and minimal toxicity toward normal cells. Acting as excellent theranostic agents targeting mitochondria, the mechanism of action of the photosensitizers has been investigated in detail in HeLa cells. The generation of cytotoxic reactive oxygen species, which has been found to be a major contributor of the coumarins’ phototoxicity, and the induction of apoptosis and/or autophagy have been identified as the cell death modes triggered after irradiation with low doses of visible light.

## Introduction

**Figure 1:**
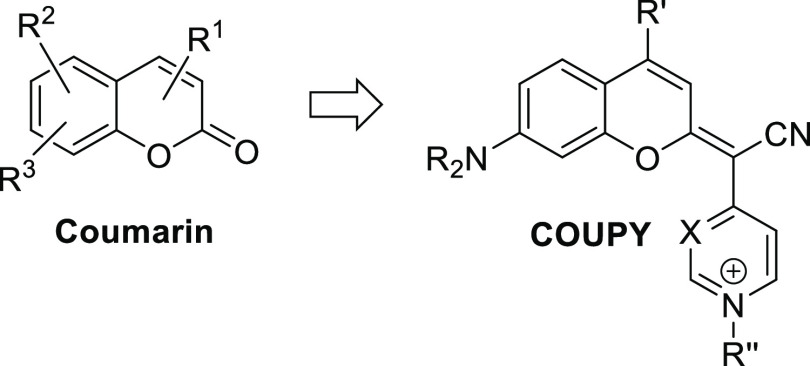
General structure of the classical coumarin scaffold and of coumarin-based COUPY derivatives.

Coumarins are a well-known family of naturally occurring molecules with a diverse range of pharmacological and biological activities owing to the privileged structure and physicochemical properties of the 2-benzopyrone moiety (Figure 1).^1,2^ Indeed, many natural and synthetic coumarins exhibit antibacterial, antiviral, antioxidant, anticoagulant, and antitumor activities, among others, and are also used in the industry as food additives and as cosmetics and perfume ingredients.^3,4^ The anticancer properties of the coumarin pharmacophore have been widely investigated, and current research efforts are dedicated to the design and development of novel coumarin analogues with the aim of addressing toxic side effects and inherent or acquired resistance of chemotherapeutic drugs in clinical use.^5−10^

Although some coumarins (e.g., psoralens) have been successfully employed for decades to treat skin disorders, such as psoriasis and vitiligo, in combination with light,^11^ this photochemotherapeutic approach requires the use of UV-A light, which has been associated with a significant increased risk of developing cutaneous melanoma.^12^ The use of coumarin derivatives as photosensitizers (PSs) in photodynamic therapy (PDT) has also drawn attention more recently, being particularly promising those compounds operating in the far-red to near infrared (NIR) region of the electromagnetic spectrum and that generate efficiently cytotoxic reactive oxygen species (ROS).^13,14^

Fluorophores based on small organic molecules have become indispensable tools to visualize cellular events as well as for the detection and/or quantification of biologically relevant species.^15,16^ Among them, fluorescent probes that can be targeted to specific organelles and operate in the optical window of biological tissues are particularly appealing because the majority of chemical and biological cell events take place inside them.^17−19^ Mitochondria are one of the most important subcellular organelles whose dysfunctions have been associated with several human pathologies, including cancer disease. Hence, mitochondria-targeted theranostic agents are highly attractive compounds for both cancer diagnosis and therapy.^20−22^ Recently, we have developed a new family of low-molecular-weight coumarins (COUPYs) in which the carbonyl group of the lactone function of the classical coumarin scaffold was replaced by the cyano(4-pyridine/pyrimidine)methylene moieties (Figure 1).^23−26^ In addition to having attractive photophysical and physicochemical properties for bioimaging and caging applications (e.g., emission in the far-red/NIR region, large Stokes’ shifts, brightness and high photostability, and aqueous solubility), N-alkylated COUPY coumarins exhibit excellent cell membrane permeability in living cells and accumulate preferentially in mitochondria and, to a lesser extent, in nucleoli and in intracellular vesicles. Mitochondria selectivity can be attributed to the lipophilic positively charged N-alkyl pyridinium/pyrimidinium moieties in the coumarin scaffold, which exploit the negative potential across the outer and inner mitochondrial membrane. In addition, conjugation of COUPY dyes to cyclometalated Ir(III) complexes allowed us to develop a new class of PSs for PDT whose mechanism of action is based on the generation of superoxide anion radicals.^27,28^ Remarkably, the COUPY derivative alone was also found to be highly phototoxic under visible-light irradiation. In such a context, COUPY coumarins hold great potential for the development of novel theranostic agents because they combine imaging and therapy in a single compound.

**Figure 2:**
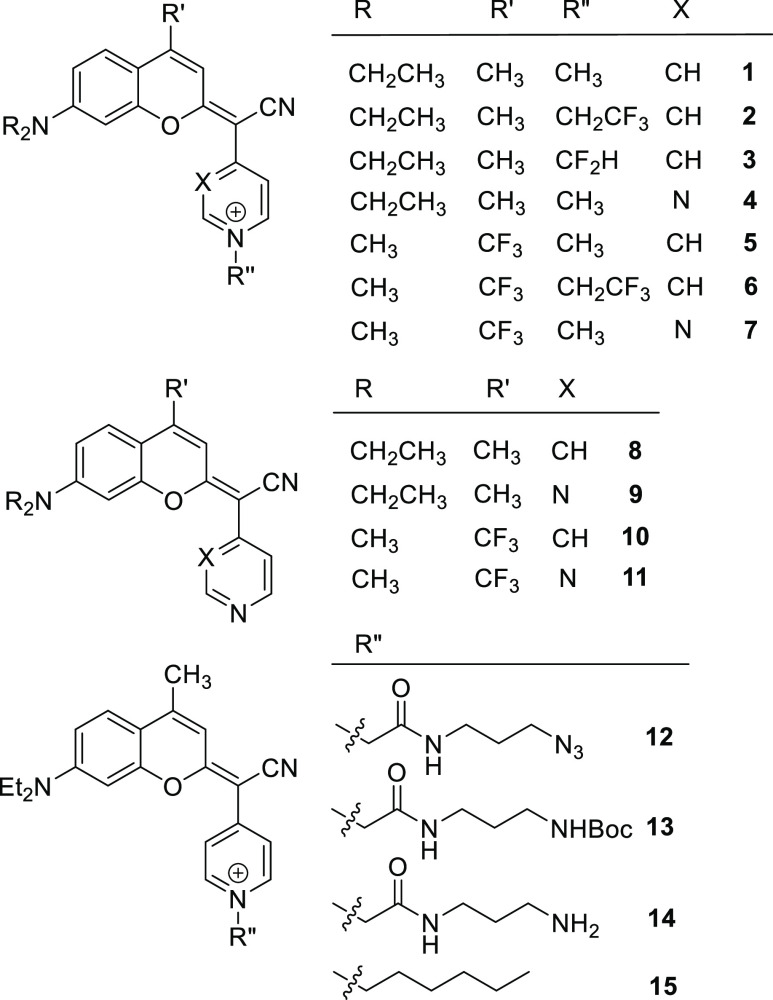
Structure of COUPY derivatives 1–15 investigated.

Herein, we have carried out a systematic study to unravel structure–activity relationships (SARSs) within the COUPY scaffold with the aim of further exploring its therapeutic value as a new anticancer agent, especially in the context of PDT. As shown in Figure 2, we have selected a small library of COUPY derivatives for biological evaluation (compounds 1–15), either cationic via N-alkylation of the heterocyclic moiety (pyridine or pyrimidine) or neutral. Cyto- and phototoxicity studies in human cancer cells as well as in nontumorigenic cells allowed us to select three hit compounds whose mechanism of action was investigated in detail. Interestingly, ROS generation was identified as a plausible major contributor of the coumarins’ phototoxicity, and depending on the structure of the compounds, apoptosis or autophagy was triggered by light irradiation.

## Results

### Synthesis and Characterization of the Compounds

**Scheme 1:**
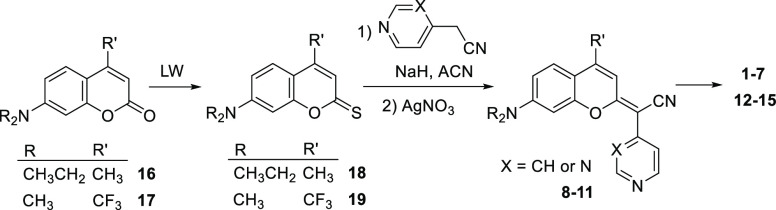
Synthesis of COUPY Derivatives 1–15 The structure of the compounds is shown in Figure 2.

Coumarins 1–14 were synthesized following previously reported procedures.^23,24^ First, the reaction of commercially available coumarins 16 and 17 with Lawesson’s reagent (LW) afforded thiocoumarins 18 and 19, respectively,^29,30^ which were condensed with 4-pyridylacetonitrile or 2-(pyrimidin-4-yl)acetonitrile to provide neutral COUPY derivatives 8–11 with excellent yields (Scheme 1). Then, the N-methylated pyridinium (1 and 5) and pyrimidinium (4 and 7) coumarins were obtained by the reaction of the corresponding precursors (8–11) with methyl trifluoromethanesulfonate in DCM at room temperature. 2,2,2-Trifluoroethyl trifluoromethanesulfonate was used as the N-alkylating reagent for synthesizing 2 and 6 from 8 and 10, respectively. *N*-Difluoromethylation of 8 with ethyl bromodifluoroacetate in a 1:1 mixture of THF/ACN at 60 °C for 24 h afforded coumarin 3.^31^ Coumarin 12 was synthesized by alkylating 8 with N-(3-azidopropyl)-2-bromoacetamide.^32^ The reaction of 8 with methyl bromoacetate followed by acidic hydrolysis and HATU-mediated coupling of N-Boc-1,3-propanediamine afforded coumarin 13, which was deprotected with HCl in dioxane to provide 14.^27^ Finally, coumarin 15 was synthesized by N-alkylation of 8 with 1-bromohexane in ACN at 60 °C for 48 h. All the compounds were purified by silica column chromatography and fully characterized by high-resolution mass spectrometry (HRMS) and ^1^H and ^13^C NMR spectroscopy. It is worth noting that all the coumarins showed an intense absorption maximum in the visible region of the electromagnetic spectrum (e.g., λ_abs_ = 543 and 595 nm for 1 and 6 in water, respectively),^23^ which allowed phototoxicity studies to be carried out with biologically compatible visible light. In addition, all COUPY derivatives showed emission in the far-red to NIR region with emission maxima in water ranging from 605 nm (1) to 683 nm (6).^23^

### Antiproliferative Activities and Phototoxicity Testing in Cancer and Normal Cells

Having at hand coumarins 1–15, we first evaluated their in vitro antiproliferative activities in human cancer cells as well as in nontumorigenic cells. For comparison, the clinical anticancer drug cisplatin was included under the same experimental conditions. As shown in Table 1, all the compounds exhibited moderate to potent cytotoxicity against cancer cells with IC_50_ values in the low micromolar range after 48 h treatment. In general, a slight reduction in the anticancer activities of the compounds toward the HeLa cell line was observed compared with those obtained for ovarian cancer cells (A2780). In addition, cytotoxicity was also evaluated in nontumorigenic ovarian tissue-derived cells (CHO) to determine differential selectivity for cancer-proliferating cells. Interestingly, the toxicity of some of the compounds in this normal cell line was much lower than that exhibited by cisplatin. Among all the tested compounds, 2 and 15 displayed higher selectivity factors (SFs) after 48 h period incubation together with potent activities against cancer cells.

**Table 1:** IC_50_ Values [μM] after 48 h Treatments with Coumarins 1–15 and Cisplatin

	HeLa	A2780	CHO	SF^a^
1	65 ± 3	16 ± 3	>100	6.3
2	9.2 ± 0.2	2.1 ± 0.1	55 ± 7	26.1
3	6.8 ± 0.3	3.8 ± 0.3	27 ± 3	7.1
4	29 ± 2	27 ± 1	>100	>3.7
5	33 ± 3	20 ± 3	>100	>5.1
6	57 ± 3	13 ± 1	37 ± 3	2.8
7	46 ± 3	1.1 ± 0.1	1.1 ± 0.1	1.0
8	42 ± 2	1.1 ± 0.3	1.4 ± 0.08	1.3
9	48 ± 3	1.0 ± 0.2	3.1 ± 0.2	3.1
10	3.5 ± 0.3	1.18 ± 0.05	1.2 ± 0.2	1.0
11	4.2 ± 0.5	2.4 ± 0.1	4.0 ± 0.5	1.7
12	17 ± 2	16 ± 1	49 ± 8	3.1
13	27 ± 2	9.9 ± 0.4	61 ± 6	6.2
14	35 ± 4	42 ± 7	>100	>2.4
15	0.19 ± 0.03	0.09 ± 0.02	1.4 ± 0.3	15.6
cisplatin	23 ± 1	2.0 ± 0.1	8.9 ± 0.4	4.5

a Selectivity factor (SF) = IC_50_(normal CHO)/IC_50_(tumoral A2780). The term “>100” indicates that no IC_50_ value was reached up to 100 μM.

HeLa cells were also selected for phototoxic activity evaluation because ideal PSs should exhibit minimal toxicity in the absence of light, and low IC_50_ values were found toward this cell line after 48 h. Photocytotoxicity was assessed via irradiation with nonharmful visible light using a light-emitting diode (LED) source covering from the cyan to the far-red region of the visible spectrum (approximately 500–700 nm) under both normoxic (21% O_2_) and hypoxic conditions (2% O_2_) according to the treatment schedule described in the Experimental Section. The IC_50_ values obtained in phototoxicity assays were used to identify the best performing compounds through determination of phototoxic indexes (PIs).

In general, as shown in Table 2, a reduction of the phototoxic effect was observed for the compounds under hypoxia. This is probably due to photodynamic reactions being restricted by low oxygen concentrations. Furthermore, the nontumorigenic renal cell line (BGM) was included in the in vitro assays to evaluate possible toxic effects in normal cells during the scheduled irradiation period. Strikingly, except for 15, none of the compounds affected cell viability of normal cells up to 100 μM under the phototoxicity procedure after 2 h incubation in the dark, which is the duration of the phototoxic procedure in cancer cells. Differential selectivity of the compounds toward cancer cells over normal cells is also reported in Table 2.

**Table 2:** Phototoxicity of the Compounds toward Cancer and Normal Cells Expressed as IC_50_ Values [μM]^a^

		HeLa		BGM^b^	
		dark	irradiated	dark	PI^c^
1	normoxia	>100	3.02 ± 0.09	>100 [1.0]	33.3
	hypoxia	>100	8.6 ± 0.3		11.6
2	normoxia	23 ± 3	2.7 ± 0.1	>100 [4.3]	8.5
	hypoxia	20 ± 3	4.7 ± 0.3		4.7
3	normoxia	>100	6.1 ± 0.3	>100 [1.0]	16.4
	hypoxia	>100	16 ± 2		6.3
4	normoxia	>100	9.5 ± 0.6	>100 [1.0]	10.5
	hypoxia	>100	47 ± 7		2.1
5	normoxia	>100	28 ± 3	>100 [1.0]	3.6
	hypoxia	>100	42 ± 4		2.4
6	normoxia	54 ± 4	20 ± 2	>100 [1.9]	2.7
	hypoxia	69 ± 4	53 ± 6		1.3
7	normoxia	24 ± 3	11 ± 1	>100 [4.2]	2.2
	hypoxia	78 ± 8	36 ± 3		2.2
8	normoxia	>100	40 ± 3	>100 [1.0]	2.5
	hypoxia	>100	>100		1.0
9	normoxia	>100	9.4 ± 0.9	>100 [1.0]	10.6
	hypoxia	>100	16 ± 1		6.3
10	normoxia	>100	41 ± 4	>100 [1.0]	2.1
	hypoxia	>100	>100		1.0
11	normoxia	51 ± 8	9 ± 1	>100 [2.0]	5.7
	hypoxia	>100	8.4 ± 1.2		11.9
12	normoxia	25 ± 2	3.4 ± 0.2	>100 [4.0]	7.4
	hypoxia	43 ± 2	9.0 ± 0.4		4.7
13	normoxia	37 ± 3	4.7 ± 0.2	>100 [2.7]	7.9
	hypoxia	>100	14 ± 2		7.1
14	normoxia	>100	9.1 ± 0.3	>100 [1.0]	11.0
	hypoxia	>100	>100		1.0
15	normoxia	2.0 ± 0.3	0.028 ± 0.004	2.2 ± 0.1 [1.1]	71.4
	hypoxia	17 ± 3	0.56 ± 0.09		30.4

a Cells were treated for 2 h (1 h of incubation and 1 h of irradiation with visible light) followed by 46 h of incubation in drug-free medium under normoxic (21% O_2_) or hypoxic conditions (2% O_2_). Control cells were left in the dark.

b Selectivity factors [i.e., SF = IC_50_ (normal BGM in dark)/IC_50_ (tumoral HeLa in dark)] are given in brackets.

c Phototoxic index (PI) = IC_50_ (dark-nonirradiated cells)/IC_50_ (irradiated cells).

### Selection of Initial Hit Compounds 1 and 2

To explore the therapeutic value of COUPY derivatives as new anticancer agents, we performed detailed SARS analysis with the aim of selecting the best coumarin candidates for further biological evaluation. First, photo and cytotoxicity of 1–7 was evaluated to assess the effect of incorporating strong electron-withdrawing groups into the coumarin scaffold, either via N-alkylation of the pyridine heterocycle or by replacing the methyl group at position 4 by CF_3_. The effect of replacing the pyridine heterocycle by pyrimidine was also investigated. Interestingly, 1–3 exhibited higher antiproliferative activities against cancer cells while displaying better SFs than 4–6 after 48 h (Table 1). A lack of selectivity over normal ovarian cells was found for 7 (SF = 1) under these conditions. Regarding phototoxicity testing under visible-light irradiation (Table 2), 1–4, which contain a CH_3_ group at the 4-position, displayed higher PI values compared to those obtained for 4-CF_3_-containing analogues (5–7).

Noticeably, the replacement of the *N*-methylpyridinium moiety by *N*-methylpyrimidinium had a negative effect on the anticancer activity under irradiation because PI values for 1 were markedly higher than those obtained for 4 in both normoxia and hypoxia. This modification led to even smaller PI values when combined with the CF_3_ group at position 4 (compound 7). Overall, these results led us to select 1 as a hit performer because this coumarin derivative exhibits a good photocytotoxic profile. Compound 2, which incorporates the 2,2,2-trifluoroethyl group at the pyridine heterocycle, was also selected on the basis of its increased preferential activity against cancer cell lines over normal cells after a long incubation period.

### Neutral Compounds Did Not Exert High Phototherapeutic Activity

Once demonstrated the good phototherapeutic activities of some of the cationic coumarins in cancer cells, the neutral parent COUPY scaffolds (compounds 8–11) were also tested to investigate the effect of the positive charge on their biological activity. Although these compounds exhibited nonselective antiproliferative action in both cancer and normal cell lines, it is worth noting that 10 and 11, which contain the CF_3_ group at position 4, exhibited higher cytotoxicity than 4-CH_3_-containing coumarins 8 and 9 against HeLa cells after 48 h (Table 1). Overall, these compounds showed lower cytotoxicity in phototoxic testing compared to 1–7 under both normoxic and hypoxic conditions. However, as shown in Table 2 the incorporation of the pyrimidine heterocycle into 9 and 11 led to higher anticancer activities after visible-light irradiation (PIs ranging from 5.7 to 11.9) than their counterparts 8 and 10, respectively, which displayed poor PIs (from 1.0 to 2.5).

### Identification of 15 as a Promising Third Hit Compound

Because the photocytotoxic profile of the COUPY pharmacophore can be modified through N-alkylation of the pyridine heterocycle, we decided to evaluate in cancer cells compounds 12–14 that incorporate N-alkyl-acetamide derivatives. Interestingly, as shown in Tables 1 and 2, a decrease in the cytotoxic activities of the three coumarins was observed after 48 h compared to the N-methylated parent compound 1 while relatively low PIs were obtained after irradiation. These results led us to increase the hydrophobicity of the coumarin by synthesizing compound 15 which incorporates an N-hexyl pyridinium moiety. Remarkably, 15 exerted a highly potent anticancer activity against cancer cells after 48 h, with IC_50_ values up to 342-fold lower than those of its parent coumarin 1 (Table 1). Moreover, although notable toxicity in normal Chinese hamster ovary (CHO) cells was observed, its SF remained higher than that of 1. Strikingly, application of low doses of visible-light irradiation greatly improved the anticancer activity of 15 because 71-fold and 30-fold increases in photopotentiation were found under normal and low-oxygen conditions, respectively (Table 2). These results led us to select coumarin 15 together with 1 and 2 as best performers for further biological experiments. Their PI values under hypoxia were comparable to those of some of the previously published reported compounds using similar protocols.^27,36^

### Intracellular Localization by Confocal Microscopy

**Figure 3:**
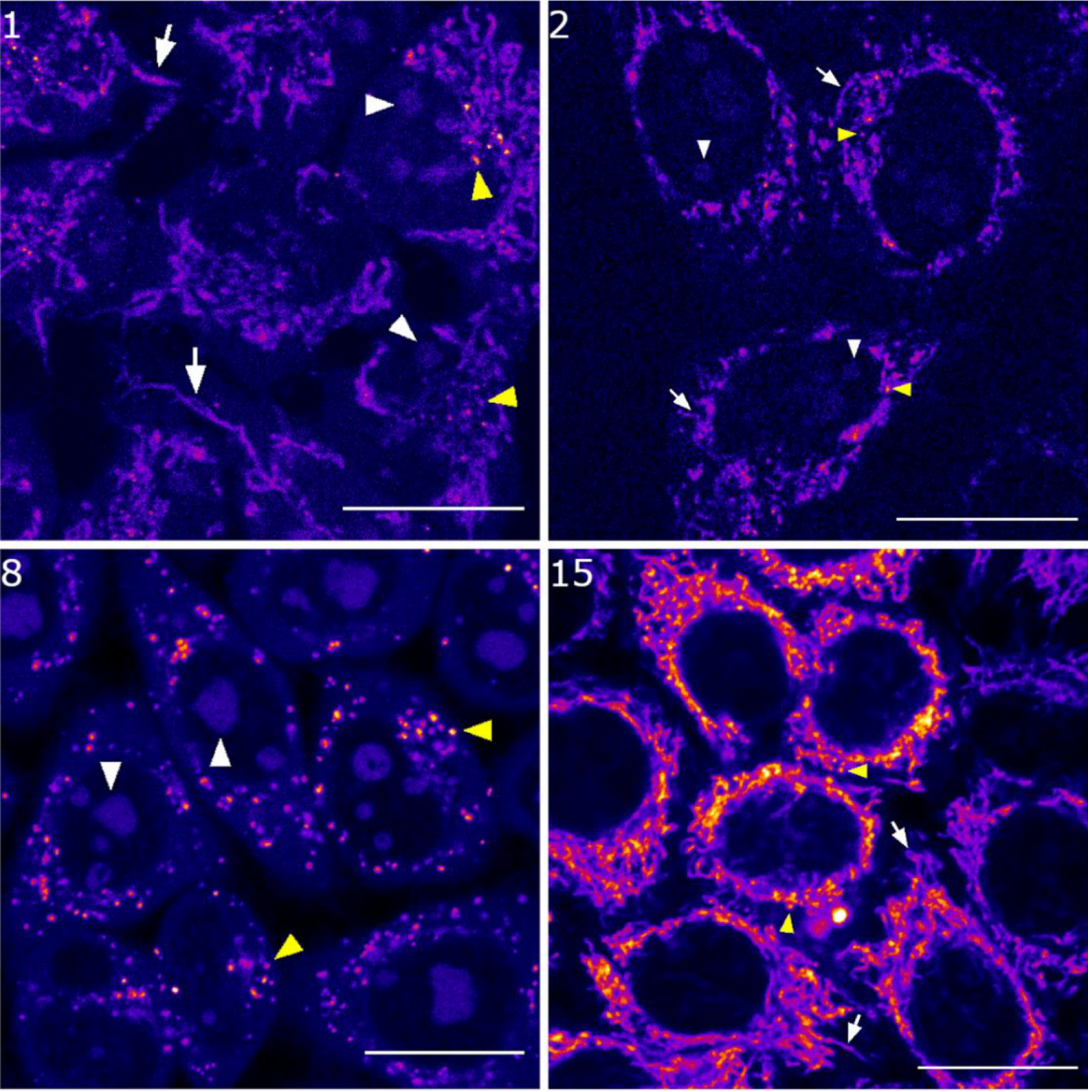
Cellular uptake of coumarins 1, 2, 8, and 15. Single confocal planes of HeLa cells incubated with the compounds (0.5 μM for 1, 1 μM for 2 and 15, and 5 μM for 8,) for 30 min at 37 °C. Excitation was performed with the 561 nm laser and emission detected from 570 to 670 nm. White arrows point out mitochondria, white arrowheads nucleoli, and yellow arrowheads vesicle staining. Scale bar: 20 μm. LUT: Fire.

Because accumulation in specific organelles might have a strong influence on the cyto- and phototoxic activity of the compounds, we next focused on evaluating the cellular uptake of some representative coumarins. Taking into account the significant differences in the anticancer activity of neutral coumarins (8–11) and of N-hexylcoumarin (15) compared with 1 and 2, we decided to investigate the cellular uptake of 8 and that of the three hit compounds (1, 2, and 15) in living HeLa cells using confocal microscopy. As shown in Figure 3, the fluorescence signal was clearly observed inside the cells in all cases after irradiation with a yellow light laser (λ_ex_ = 561 nm), which confirmed an excellent cellular uptake of the compounds. However, the overall pattern of staining was slightly different when comparing them. As previously found, coumarins 1 and 2 accumulated preferentially in mitochondria, although nucleoli and some intracellular vesicles were also stained (Figure 3). By contrast, coumarin 8 accumulated mainly in nucleoli as well as in vesicles, but it did not accumulate in mitochondria (Figure 3). The absence of a positive charge might facilitate accumulation in nucleoli, probably through intercalation between base pairs in RNA. On the other hand, coumarin 15 accumulated in mitochondria but not in nucleoli (Figure 3). To our surprise, irradiation of the cells with the excitation laser beam triggered important changes in the morphology of mitochondria (Figure S4 and supplementary video). The characteristic donut-shaped morphology observed in all the cells is indicative of mitochondrial stress^33^ and could be related to ROS generation.

### Generation of ROS in HeLa Cells after Photoirradiation

**Figure 4:**
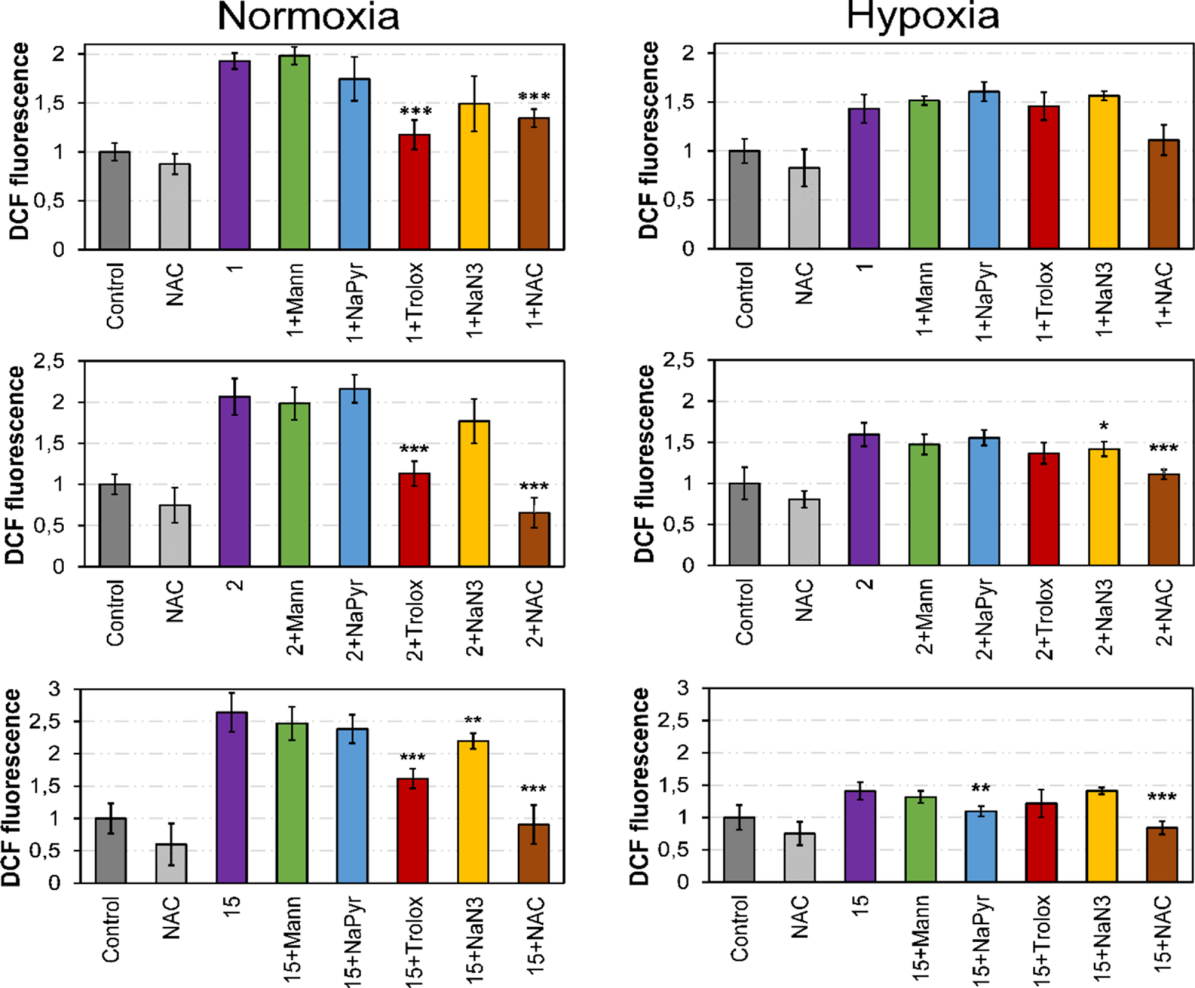
ROS levels in HeLa cells upon irradiation treatments with 5 μM of compounds 1, 2, and 15 (1 h in the dark followed by 1 h under light irradiation). Statistical significance from treated cells based on *p < 0.05, **p < 0.01, and ***p < 0.001 using the unpaired t-test. Data represented as mean ± SD (*n* = 3 replicates).

Central to the PDT is the generation of ROS derived from photochemical reactions involving the excited state of PSs under light irradiation.^34^ However, one of the main drawbacks of PDT is related to a strong dependence on molecular oxygen to produce toxic ROS,^34^ which presents difficulties for the treatment of hypoxic tumors. Therefore, we decided to determine ROS generation under both normal and low-oxygen conditions to gain insights into the underlying phototherapeutic mechanisms of the hit compounds. As shown in Figure 4 and Figures S5 and S6, when irradiated under normoxia, intracellular ROS levels increased in HeLa cells treated with coumarins compared to control irradiated cells with a 2-fold change for 1 and 2 and 2.5-fold for 15. These results correlate with data obtained in phototoxicity studies (Table 2) and especially for 15, which showed potent phototoxic activity and was demonstrated to induce the highest production of ROS after light irradiation. In contrast, hypoxic conditions slightly diminished the photoinduced generation of ROS in cancer cells, showing a similar correlation with phototoxicity values.

Further analysis was performed to elucidate the main ROS involved. In addition to singlet oxygen (^1^O_2_), which is produced in type II photochemical reactions, type I processes involve the generation of cytotoxic ROS such as superoxide anions (^•^O_2_^–^), hydroxyl radicals (^•^OH), hydrogen peroxide (H_2_O_2_), and peroxyl radicals (ROO^•^).^35^ To investigate the type of intracellular ROS produced after irradiation in the presence of coumarins 1, 2, and 15, HeLa cells were incubated first with selective scavengers for each type of ROS and then probed with ROS-sensitive dyes using previously described methodologies.^27,36^ The scavengers used included tiron (5 mM) for ^•^O_2_^–^, d-mannitol (Mann; 50 mM) for ^•^OH, sodium azide (NaN_3_; 5 mM) for ^1^O_2_, sodium pyruvate (NaPyr; 10 mM) for H_2_O_2_, and Trolox (0.1 mM) for ROO^•^ species. As shown in Figure 4, cotreatment with general scavenger *N*-acetyl-cysteine (NAC; 5 mM) partially attenuated ROS production in all coumarin-treated cells under both normoxia and hypoxia.

Under normoxic conditions, only the use of Trolox, which prevents the formation of peroxyl radical (ROO^•^), managed to reduce significantly ROS generation in 1, 2, and 15-treated cells after irradiation. However, under hypoxia, different ROS were identified for each compound after light irradiation. On the one hand, none of the scavengers significantly prevented the formation of ROS upon treatment with 1 under hypoxia, which rendered some difficulties to identify specific ROS involved using this methodology. Because coincubation with Trolox and NaN_3_ decreased ROS levels, ^1^O_2_ species seemed to be involved in 2-treated cells upon irradiation. For 15, addition of the ^1^O_2_ scavenger (NaN_3_) also diminished ROS production under normoxia, whereas the use of the H_2_O_2_ scavenger (NaPyr) prevented ROS production under hypoxia.

The ability for coumarins to produce superoxide anions (^•^O_2_^–^) was also investigated using a dihydroethidium (DHE) probe. Under normal oxygen conditions, no increase in DHE fluorescence was observed after irradiation (Figure S7). However, 1 and 2 (but not 15) slightly induced ^•^O_2_^–^ under low-oxygen conditions compared to irradiated control cells. This ROS generation was not reverted by the use of superoxide scavenger tiron.

Furthermore, the singlet oxygen generation by COUPY derivatives 1, 2, and 15 was investigated in a cell-free assay using 1,3-diphenylisobenzofuran (DPBF) as an ^1^O_2_ scavenger and methylene blue as a reference (Φ_Δ*s*_ = 0.57 in DCM).^37−39^ DPBF is a green fluorescent probe that decomposes into a colorless product upon reaction with singlet oxygen.^40^ The decrease in the intensity of the absorption band of DPFB at 411 nm as a result of the reaction with singlet oxygen in an air-saturated DCM solution was monitored upon excitation with a high-power LED source of green light (505 nm, 100 mW cm^–2^).^26^ As shown in Figures S8 and S9, a gradual decrease in the absorbance of DPBF at 411 nm was observed upon irradiation in the presence of COUPY derivatives, being much more pronounced in the case of coumarin 15. In good agreement with cellular experiments, the highest efficacy of singlet oxygen production was obtained for coumarin 15 (Φ_Δ_ = 0.11), whereas lower yields were found for 1 and 2 (Φ_Δ_ = 0.049 and 0.046, respectively).

### Mitochondrial Potential Was Depleted by Treatment with 15

Once mitochondrial localization of the three hit coumarins was revealed by confocal microscopy, we evaluated mitochondrial membrane potential (MMP) (Figure S10) using JC-1 dye as its depolarization is a hallmark of mitochondrial dysfunction.^20^ Treatment with 1 and 2 did not affect MMP, whereas 15 induced a great population of cells with depleted MMP after 24 h; flow cytometry dot plots resemble those treated with the electron transport chain inhibitor antimycin A.

### Compounds 1 and 15 Induced Autophagy after Photoirradiation

**Figure 5:**
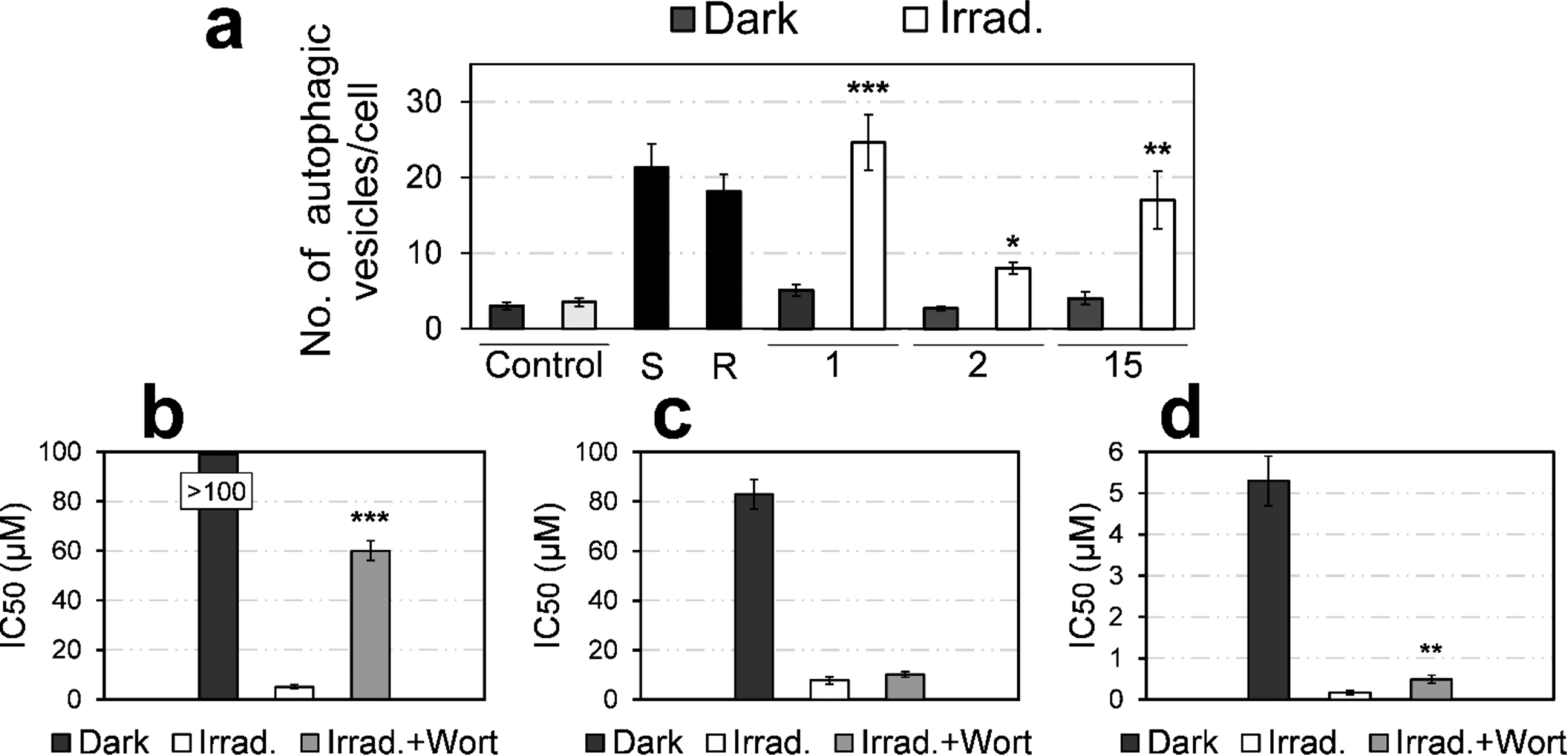
(a) Number of autophagic vesicles in HeLa cells after irradiation treatments as quantified by confocal microcopy imaging through monodansylcadaverine (MDC) staining. (S: Starvation for 2 h; R: resveratrol 50 μM). Data represented the mean ± SD from >10 cells from two independent experiments. (b–d) IC_50_ values (mean ± SD) in HeLa cells for 1, 2, and 15, respectively, in the dark, after irradiation (0.5 h incubation, 1 h irradiation with visible light, and 48 h of recovery) or pretreated with wortmannin (50 μM) for 1 h prior irradiation schedule. Statistical significance from irradiation treatments based on *p < 0.05, **p < 0.01, and ***p < 0.001 using the unpaired t-test.

Next, we decided to investigate if autophagy was induced after irradiation treatments with coumarins under a confocal microscope after labeling of intracellular autophagic vacuoles with MDC dye. Both starvation and chemically induced autophagy with resveratrol served as positive controls for autophagy. Notably, when irradiated, autophagic vacuoles were formed upon treatment with 1 and 15 as revealed by MDC staining (Figures S11 and S12). Quantitative analysis revealed that irradiation treatments with these coumarins increased the number of MDC-labeled vesicles 3 to 5 times compared to nonirradiated samples, thus displaying a similar situation to that produced by starvation or resveratrol treatment (Figure 5a). However, irradiation by visible light did not induce such a number of autophagy processes in cells in the presence of 2.

To confirm that autophagy initiation was related to cell viability upon irradiation with the hit compounds, the autophagy inhibitor wortmannin, which selectively blocks PI3K enzyme and autophagosome formation,^41^ was used. As seen in Figure 5b–d, pretreatment with wortmannin significantly attenuated the cytotoxic effect of 1 and 15 but not 2 after light application, thus corroborating autophagy induction being produced for these coumarins.

### Hit Compounds Increased SubG1-Phase Populations in Cancer Cells

In order to gain insights into the mechanism of action of COUPY coumarins, cell cycle distribution of HeLa cells was evaluated in the dark and after light irradiation. Although in the dark 1 and 2 did not cause apparent changes in cell cycle progression at 5 μM, upon light irradiation, the G1-phase population decreased and a concomitant increase of subG1-phase cells was observed (Figures S13 and S14). A similar situation occurred with 15 treatment, which resulted in accumulation of subG1-phase populations only after irradiation with visible light. As the subG1 phase is indicative of DNA fragmentation, additional flow cytometry experiments were performed to discriminate if this population was due to apoptosis or necrosis.

### Apoptosis Was Induced by Hit Compounds

To check whether these subG1 populations corresponded to apoptosis or necrosis, dual Annexin V/propidium iodide labeling experiments were performed, which allowed the detection of four populations, *that is*, viable cells, necrotic cells, and early- and late-stage apoptosis. Apart from DNA fragmentation, apoptosis at the early stage is characterized by changes in the symmetry of phospholipids in the cytoplasmic membrane, whereas the late stage involves the disruption of the membrane integrity, thus allowing propidium iodide to enter. The cell membrane of necrotic cells, in contrast, becomes readily permeable to propidium iodide but does not involve phospholipid translocations.

**Figure 6:**
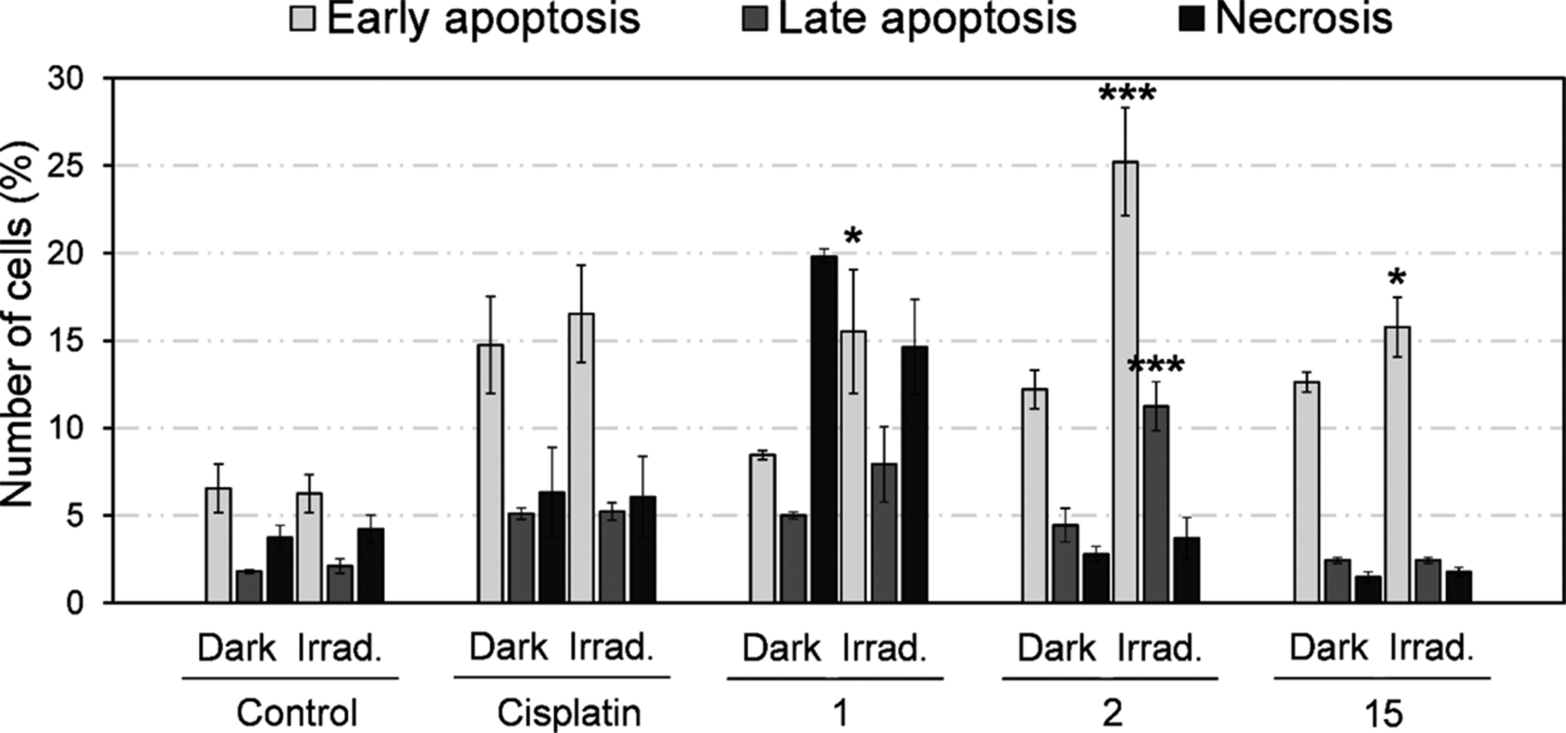
Flow cytometry evaluation of cell death induction in HeLa cells upon treatment with coumarins (5 μM for 1 and 2; 0.5 μM for 15) after irradiation treatments as revealed by dual Annexin V/propidium iodide labeling. Cisplatin (30 μM) used as a positive control for cell death induction. Data represented as mean ± SD and statistical significance from dark/irradiated conditions based on *p < 0.05, **p < 0.01, and ***p < 0.001 obtained using the unpaired t-test.

Treatment with 1 (5 μM) in the dark increased the number of cells in necrosis (propidium iodide positive quadrant), but upon irradiation, early apoptosis was significantly promoted (Annexin V positive quadrant), thus indicating that 1 was able to photoinduce apoptosis (Figure 6 and Figure S15). Similarly, 15 at 0.5 μM induced apoptosis both in the dark, and after irradiation, the population in early apoptosis significantly increased after light treatment. In contrast, 2 induced a small population of cells to apoptosis at 5 μM, but upon irradiation, this population was very significantly increased in both early and late apoptotic phases. Overall, phototreatment with 2 managed to induce higher apoptotic levels than 1 and 15 (Figure 6).

## Discussion and Conclusions

The evaluation of coumarin derivatives 1–15 in ovarian and cervical cancer cells as well as in nontumorigenic ovarian and renal cells permitted establishing a preliminary SARS rationale that enabled the identification of new PSs based on COUPY scaffolds with promising anticancer photoactivities. On the basis of cyto- and phototoxic activity determination (Tables 1 and 2), coumarins 1, 2, and 15 were first selected as hit compounds for further biological evaluation because they exhibited high antiproliferative activities in cancer cells, good phototherapeutic indexes after visible light irradiation, and minimal toxicity toward normal dividing cells. In general, the lower photopotentiation of the coumarins under hypoxic conditions compared with normoxia suggested that photodynamic reactions involving molecular oxygen were required for the phototoxicity of the compounds. Indeed, hit compounds managed to increase up to 2.5-fold the production of cytotoxic ROS after irradiation under normoxic conditions compared to irradiated control cells, suggesting that their photoactivity resulted from PDT reactions (Figures S5 and S6). In contrast, the photoinduced generation of ROS in cancer cells under hypoxic conditions was slightly diminished. These results were correlated with those found for quantum yield of singlet oxygen formation by COUPY derivatives 1, 2, and 15 in DPBF cell-free assays (Figures S8 and S9), thus confirming their main mode of action after light irradiation.

Once ROS generation was identified as a plausible major contributor of the coumarins’ phototoxicity, we decided to explore the use of selective ROS scavengers to discriminate the type of cytotoxic oxygen species involved (Figure 4). In general, only the use of Trolox under normoxia prevented ROS generation after treatment of the cells with the three hit coumarins, which indicated that peroxyl radicals (ROO^•^) might be produced after irradiation. These results suggest that the COUPY scaffold might specifically induce the generation of this type of ROS after application of low doses of visible light. Particularly for 1, under hypoxia, none of the scavengers significantly diminished ROS production, thus suggesting that either various types of ROS might be acting at the same time under low-oxygen conditions or that the methodology based on the use of scavengers was not sensitive enough to identify all the specific ROS produced. However, for 2 and 15, singlet oxygen (^1^O_2_) species seemed to be involved under hypoxia because coincubation with the NaN_3_ scavenger significantly decreased ROS levels. In addition, superoxide anions appeared to be slightly generated under hypoxia in the case of 1 according to DHE probe although the tiron scavenger could not prevent its formation, suggesting additional types of ROS implied (Figure S7). Altogether, these results indicate that different PDT reactions may take place depending on the oxygen concentration in which cells are growing, with type I ROO^•^ producing reactions predominating under normoxia and ^1^O_2_ being raised under hypoxia. These differences in ROS generation could be partially responsible for the chemoresistance observed in hypoxic cells. PSs operating at low oxygen concentrations are particularly appealing for treating deep-seated hypoxic tumors in the clinics.

Confocal microscopy studies revealed that COUPY derivatives can be used to target specific organelles within cancer cells thanks to their excellent cell plasma and nuclear membrane permeability, their fluorescence emission being in the far-red to NIR region easily detected after incubation at low concentrations for a short period of time. Interestingly, depending on the coumarin, different localizations inside HeLa cells were observed, thus indicating that the structure of these compounds can be fine-tuned to selectively act as probes for specific sites within living cells. Indeed, neutral coumarin 8 accumulated preferentially in nucleoli, whereas the positively charged N-hexyl pyridinium coumarin 15 was localized in mitochondria (Figure 3). By contrast, coumarins 1 and 2 accumulated in both organelles, although preferably in mitochondria (Figure 3). The mitochondria accumulation of the three hit coumarins (1, 2, and 15) in cancer cells led us to hypothesize that mitochondrial dysfunction could be triggered after treatment with these coumarins. To test this hypothesis, we evaluated the MMP of living cancer cells, finding that 15 but not 1 nor 2 managed to induce mitochondrial depolarization (Figure S10), suggesting that the N-alkylated hexyl group had a crucial effect regarding mitochondrial membrane polarization. These results are in good agreement with confocal microscopy observations which revealed degeneration of the mitochondria (Figure S4).

Because photodynamic damage of organelles like mitochondria can trigger various types of cell death including autophagy, apoptosis, or paraptosis,^42^ additional cell-based experiments were performed to elucidate the mechanism of action of hit compounds after irradiation.

On the one hand, autophagy is a self-digestion process by which cells degrade and renew damaged organelles.^43^ Although autophagy might play opposite roles regarding cancer cell fate, as it participates in either cytoprotection or cell death, mounting evidence has shown that photodamage can lead to autophagy-associated cellular death.^44,45^ Confocal microscopy imaging using MDC dye revealed autophagic processes being initiated after treatment with 1 and 15 after light irradiation but not with 2 (Figure 5 and Figures S11 and S12), which led us to think that the N-alkyl pyridinium moiety of the coumarin scaffold might be a key modulator for autophagy induction (coumarin 2 incorporates a strong electron-withdrawing CF_3_ group). The role of autophagy in cell viability was further confirmed by pretreatment with autophagic inhibitor wortmannin, which effectively attenuated the phototoxic action of 1 and 15, thereby corroborating autophagy as a cell death mechanism. The ability of both hit compounds to accumulate in active mitochondria, together with the formation of autophagic vesicles after irradiation, prompted us the idea of mitophagy being produced, although further research will be needed to elucidate this hypothesis.

On the other hand, the initiation of autophagy by coumarin PSs could be understood as an adaptive response of cells to treatments, which would activate cytoprotective pathways leading to removal of damaged organelles. However, it is known that, beyond a certain threshold, the organellar stress (induced in these cases by phototreatment) could then cause apoptosis.^46^

Apoptosis is a well-known type of programmed cell death characterized by large-scale DNA fragmentation.^47^ Flow cytometry experiments with coumarins after light irradiation showed an increase of fragmented DNA observed as subG1 populations of HeLa cells (Figure S13). This was further confirmed to correspond to early and late apoptosis being induced rather than necrosis (Figure 6). Strikingly, treatment with 2 after irradiation, which did not result in autophagy induction, provoked broader populations of apoptotic cells than 1 and 15, which did initiate autophagic processes. Altogether, these results indicated that 1 and 15 were able to induce both autophagic and apoptotic cell death upon irradiation, whereas treatment with 2 only resulted in apoptosis, thus suggesting that the 2,2,2-trifluoroethyl group of the coumarin moiety could be responsible for the differences in cell death mechanisms.

In summary, a small library of coumarin derivatives has been synthesized, and their applicability as new PSs for PDT was explored. The determination of the in vitro cyto- and phototoxicity in cancer and normal cells, which has revealed important SARSs, led to the identification of three hit candidates (compounds 1, 2, and 15) because of their good phototherapeutic outcomes (with a PI higher than 71 for 15). Our results showed that PDT reactions involving specific cytotoxic ROS (i.e., peroxyl radicals in normoxia and singlet oxygen in hypoxia) were predominantly generated in cancer cells under visible-light irradiation. Importantly, the fluorescence properties of COUPY coumarins allowed us to confirm rapid cellular uptake and preferential accumulation in mitochondria, and flow cytometry experiments confirmed that coumarin 15 induced depolarization of MMP, which can be attributed to the N-alkylated hexyl group. In addition, 1 and 15 were able to promote both apoptotic cell death and autophagy induction after visible-light irradiation, whereas 2 only resulted in apoptosis being induced. The high anticancer activities found under both normoxia and hypoxia in the presence of nonharmful visible light along with the excellent bioimaging properties of COUPY coumarins make them promising PDT theranostic candidates for potential phototherapy of solid cancers. Work is in progress in our laboratory to develop novel PSs based on COUPY scaffolds with operability in the phototherapeutic window, especially in the NIR region, to facilitate clinical applications.

## Experimental Section

### Chemistry

Unless otherwise stated, common chemicals and solvents (HPLC grade or reagent grade quality) were purchased from commercial sources and used without further purification. Aluminum plates coated with a 0.2 mm thick layer of silica gel 60 F254 were used for thin-layer chromatography (TLC) analyses, whereas flash column chromatography purification was carried out using silica gel 60 (230–400 mesh). Nuclear magnetic resonance (NMR) spectra were recorded at 25 °C in a 400 MHz spectrometer using the deuterated solvent as an internal deuterium lock. Tetramethylsilane was used as an internal reference (0 ppm) for ^1^H spectra recorded in CDCl_3_ and the residual protic signal of the solvent (77.16 ppm) for ^13^C spectra. Chemical shifts are reported in part per million (ppm) in the δ scale, coupling constants in Hz and multiplicity as follows: s (singlet), d (doublet), t (triplet), q (quartet), m (multiplet), dd (doublet of doublets), and br (broad signal). High-resolution electrospray ionization mass spectra (ESI-MS) were recorded on an instrument equipped with a single quadrupole detector coupled to a high-performance liquid chromatography (HPLC) system. The purity of final compounds was determined by reversed-phase HPLC analyses on a Jupiter Proteo C12 column (250 × 4.6 mm, 90 Å 4 μm, flow rate: 1 mL/min) using linear gradients of 0.1% formic acid in MilliQ H_2_O (A) and 0.1% formic acid in ACN (B). The HPLC column was maintained at 25 °C. All final compounds were >95% pure by this method.

Coumarins 1–14 were synthesized following previously reported procedures^23−25^ Coumarin 15. 1-Bromohexane (600 μL, 4.3 mmol) was added to a solution of coumarin 8 (150 mg, 0.45 mmol) in anhydrous ACN (15 mL). The mixture was stirred for 48 h at 60 °C under an Ar atmosphere and protected from light. The crude product was evaporated under reduced pressure and purified by column chromatography (silica gel, 0–5% MeOH in DCM) to give 187 mg of the bromide salt of 15 as a pink solid (yield 84%). TLC: *R*_f_ (5% MeOH in DCM) 0.26. ^1^H NMR (400 MHz, CDCl_3_) δ: (ppm) 8.76 (2H, d, *J* = 7.2 Hz), 8.35 (2H, d, *J* = 7.2 Hz), 7.50 (1H, d, *J* = 9.2 Hz), 7.43 (1H, br), 6.89 (1H, br), 6.77 (1H, dd, *J* = 9.2, 2.4 Hz), 4.43 (2H, t, *J* = 7.2 Hz), 3.67 (4H, q, *J* = 7.2 Hz), 2.50 (3H, s), 1.97 (2H, m), 1.30 (14H, m). 0.88 (3H, t, *J* = 7.2 Hz). ^13^C NMR (101 Hz, CDCl_3_) δ: (ppm) 167.6, 155.7, 152.9, 152.2, 149.9, 142.5, 126.2, 121.6, 118.7, 112.0, 111.1, 110.8, 98.2, 79.1, 60.0, 45.5, 31.5, 31.3, 26.0, 22.5, 19.1, 14.0, 12.8. HRMS (ESI), positive mode: *m/z* 416.2698 (calcd mass for C_27_H_34_N_3_O [M]^+^ 416.2696).

### Cell Culture and Cell Lines

The human ovarian cancer cell line, A2780, was cultured in RPMI-1640 cell medium supplemented with 10% fetal bovine serum (FBS) and 2 mM l-glutamine; CHO cells were grown in F-12 cell medium with 10 FBS and l-glutamine; human cervix adenocarcinoma cells, HeLa, and buffalo green monkey kidney cells, BGM, were maintained in Dulbecco’s modified Eagle medium (DMEM) supplemented with 10% FBS, l-glutamine, 1% penicillin–streptomycin, and 1% nonessential amino acids. Cells were cultured in a humidified incubator at 310 K in a 5% CO_2_ atmosphere and subcultured twice a week with appropriate densities and were confirmed to be mycoplasma-free using a standard Hoechst DNA staining method.

### Photo and Cytotoxic Activity Determination

Briefly, A2780, HeLa, CHO, and BGM cells were maintained under appropriate conditions and cultured in 96-well plates at a density of 5000 cells/well in complete medium and incubated for 24 h. Serial dilutions of the compounds were added at the final concentrations in the range of 0 to 100 μM in a final volume of 100 μL per well. For cytotoxicity studies, a treatment period of 48 h was allowed. For phototoxicity studies in HeLa cells, the light-based treatment schedule was performed as follows: 1 h incubation with the compound in the dark followed by 1 h incubation under irradiation conditions by placing the Photoreactor EXPO-LED from Luzchem (Canada) fitted with white lamps (final light intensity applied of 2.95 mW/cm^2^ at λ_max_ = 520 nm; 2.6 mW/cm^2^ at λ_max_ = 595 nm) inside the CO_2_ incubator. A detailed setup and methodologies for photocytotoxicity experiments under hypoxia conditions are reported in the Supporting Information (Figure S16). Then, drug-containing media were removed, and fresh media were added for a 48 h cell recovery period; the temperature throughout the experiment remained at 310 K. Once the recovery period completed, the medium was aspirated by suction, and cells were loaded with 50 μL of MTT solution for 4 h and DMSO solubilization before reading absorbance on a microplate reader (FLUOstar Omega). Data from dose–response sigmoidal curves were processed with SigmaPlot 14 software to calculate IC50 values (*n* = 3 replicates).

### Fluorescence Imaging

HeLa cells were maintained in DMEM containing high glucose (4.5 g/L) and supplemented with 10% fetal calf serum and 50 U/mL penicillin–streptomycin. For cellular uptake experiments and posterior observation under the microscope, cells were seeded on glass-bottom dishes (P35G-1.5-14-C, Mattek). Twenty four hours after cell seeding, cells were incubated for 30 min at 37 °C with coumarins (0.5 μM for 1; 1 μM for 12 and 15; 5 μM for 8) in supplemented DMEM. Then cells were washed three times with DPBS (Dulbecco’s phosphate-buffered saline) to remove the excess of the fluorophores and kept in low-glucose DMEM without phenol red for fluorescence imaging.

All microscopy observations were made using a Zeiss LSM 880 confocal microscope equipped with a 561 nm laser. The microscope was also equipped with a Heating Insert P S (Pecon) and a 5% CO_2_ providing system. Cells were observed at 37 °C using a 63 × 1.2 glycerol immersion objective. All the compounds were excited using the 561 nm laser and detected from 570 to 670 nm. Image processing and analysis were performed using Fiji.^48^

### ROS Determination

ROS were determined using the 2′,7′-dichlorofluorescein diacetate (DCFH-DA) and DHE reagents. HeLa cells were seeded onto 96-well plates at 2 × 10^4^ cells/well for 24 h under normoxia (21% O_2_) or hypoxia (2% O_2_) in the humidified CO_2_ incubator. Cells were then cotreated with selective ROS scavengers and with 5 μM of the tested complexes for 1 h. NAC (5 mM) was used as a general scavenger for ROS production. Formation of ^1^O_2_ was prevented using sodium azide (NaN_3_) at a final concentration of 5 mM; hydroxyl radicals (^•^OH) were scavenged using d-Mannitol (Mann) at 50 Mm; superoxide anion (^•^O_2_^–^) production was reduced using the tiron scavenger (5 mM); generation hydrogen peroxide was prevented using sodium pyruvate (NaPyr) at 10 mM; and peroxyl radical (ROO^•^) species were scavenged using 0.1 mM of Trolox. The ROS scavengers remained throughout the experiment. After treatment application, cells were incubated for 1 h in the dark followed by 1 h of irradiation with visible light. After irradiation, the cells were stained using 10 μM of DCFH-DA or, alternatively, DHE for 30 min. Cells were then trypsinized to allow cell capture by the flow cytometer (Fortessa X20) using the 96-well plate adaptation and analyzed using Flowing Software version 2.5.1. The assay was performed at least in two independent experiments (*n* = 3 per replicate).

### MMP Assessments

MMP was evaluated with the fluorescent probe JC-1 chloride (Promocell). HeLa cells at a density of 1.5 × 10^5^ were seeded for 24 h in complete medium on 12-well plates and then treated with indicated concentrations of tested complexes for 24 h. Untreated cells contained maximal concentration of DMSO used in the treatment (0.4%) and were used as a negative control, whereas antimycin A (50 μM) was used as a positive control for mitochondrial dysfunction. After drug exposure, the cells were incubated with JC-1 dye (1 μM) for 20 min and subjected to flow cytometry (FACSCAlibur BecktonDickinson; 10^4^ events acquired per sample), using λ_exc_ = 488 nm, λ_em_ = 530 ± 30 nm (green), and 585 ± 30 nm (red) parameters to discriminate green JC1 monomers (FL1-H channel) and red JC1 aggregates (FL2-H channel). At least two independent experiments were performed (*n* = 2).

### Autophagy Evaluation

Autophagy induction was evaluated using the fluorescent probe MDC (Sigma) as described elsewhere.^49^ Briefly, HeLa cells at a density of 1.5 × 10^4^ cells/cm^2^ were seeded onto confocal 8 μ-slide chambers (Ibidi) and allowed to attach and grow inside the CO_2_ incubator. Cells were then treated with equitoxic concentrations (close to IC_50_ under light, i.e., 5 μM for 1 and 2; 0.5 μM for 15) of tested compounds following phototoxicity schedules as described (0.5 h incubation +1 h irradiation). Two positive controls for autophagy were used: starvation, which was induced by replacing complete media to EBSS saline solution for 2 h, and chemical induction with resveratrol (50 μM, 2 h).^50^ After irradiation, drug-containing media were replaced by fresh media, and 6 h cell recovery period was allowed prior imaging. Cells were then washed with PBS, stained with the selective autophagy marker MDC (0.05 mM in PBS) for 10 min at 310 K, and washed again three times with PBS. Confocal microscopy images were obtained with SP8 Leica systems (λ_exc_ = 405 nm). Experiments were repeated twice, and the number of MDC vacuolation was processed from at least 10 cells from the two data sets of experiments using Fiji software.

### Cell Cycle Distribution Analysis

HeLa cells were seeded into 12-well plates at a density of 1.5 × 10^5^ cells/well. Compounds and cisplatin were added following the described treatment schedule (0.5 h incubation +1 h irradiation). After 24 h of the cell recovery period, cells were trypsinized and fixed in ice-cold ethanol 70% in PBS for 1 h. After centrifugation, a staining solution containing 40 μg/mL PI and 1 μg/mL RNase was added for 30 min at 310 K, and the samples were subjected to analysis using a FACSCalibur cytometer (λ_exc_ = 488 nm and λ_em_ = 630). At least two independent experiments were performed (*n* = 3) as measured in the FL2-A channel.

### Cell Death Induction Assays

Cell death induction of the cells was evaluated using the FITC-Annexin V/propidium iodide labeling method. Briefly, HeLa cells were seeded in 12-well plates at a density of 1.5 × 10^5^cells/well and incubated overnight. Compounds and cisplatin were added following the described treatment schedule (0.5 h incubation + 1 h irradiation). After 24 h of the cell recovery period, cells were harvested by trypsinization, washed with PBS, and centrifuged, and the pellets were resuspended in 200 μL binding buffer. Then, Annexin-V-FLUOS and propidium iodide were added as instructed by the manufacturer (Roche). The resuspended cell solution was left at room temperature in the dark for 15 min prior to analysis by flow cytometry (FACSCalibur BecktonDickinson; 10^4^ events acquired per sample), registering at 620 and 525 nm for propidium iodide and Annexin V, respectively, λ_exc_ = 488 nm using FL1 and FL2 channels. Data were analyzed using FlowingSoftware version 2.5.1. The assay was performed at least in two independent experiments (*n* = 3).

## Supporting Information Available

Additional figures for NMR and MS characterization of coumarin 15, fluorescence imaging, singlet oxygen measurements, and biological studies (ROS generation in HeLa cells, superoxide anion generation in HeLa cells after irradiation, mitochondrial potential assessment, autophagy induction, and cell cycle distribution experiments) (PDF)
Fluorescence imaging video of HeLa cell-incubated coumarin 15 (AVI)
Molecular formula strings (CSV)

## Supplementary Material

jm1c01254_si_001.pdf

jm1c01254_si_002.avi

## Abbreviations

ACN: acetonitrile
CHO: Chinese hamster ovary
DCM: dichloromethane
DHE: dihydroethidium
ESI: electrospray ionization
HRMS: high-resolution mass spectrometry
LW: Lawesson’s reagent
MDC: monodansylcadaverine
MMP: mitochondrial membrane potential
NAC: *N*-acetylcysteine
NMR: nuclear magnetic resonance
PDT: photodynamic therapy
PI: phototoxic index
PS: photosensitizer
ROS: reactive oxygen species
THF: tetrahydrofuran
TLC: thin-layer chromatography
