# Supplementary material for: COUPY Coumarins as Novel Mitochondria-Targeted Photodynamic Therapy Anticancer Agents
Source: J Med Chem. 2021 Nov 19;64(23):17209–20. doi: 10.1021/acs.jmedchem.1c01254 (PMC8667040; doi:10.1021/acs.jmedchem.1c01254)
Supplement: Supplementary file 1 — jm1c01254_si_001.pdf [file jm1c01254_si_001.pdf]

SUPPLEMENTARY INFORMATION

COUPY coumarins as novel mitochondria-targeted PDT anticancer agents

Enrique Ortega-Forte[1], Anna Rovira[2], Albert Gandioso[2], Joaquín Bonelli[2], Manel Bosch[3], José Ruiz[1,*], Vicente Marchán[2,*].

[1] Departamento de Química Inorgánica, Universidad de Murcia and Institute for Bio-Health Research of Murcia (IMIB-Arrixaca), Campus de Espinardo, E-30071 Murcia (Spain)

[2] Departament de Química Inorgànica i Orgànica, Secció de Química Orgànica, IBUB, Universitat de Barcelona Martí i Franqués 1–11, E-08028 Barcelona (Spain).

[3] Unitat de Microscòpia Òptica Avançada, Centres Científics i Tecnològics, Universitat de Barcelona, Av. Diagonal 643, E- 08028 Barcelona (Spain)

E-mail: vmarchan@ub.edu, jruiz@um.es

Table of contents

1.- ^{1}H and 13C NMR spectra and HR ESI-MS of coumarin 15 S2

2.- Reversed-phase HPLC analysis of hit coumarins 1, 2 and 15 S3

3.- Fluorescence imaging S4

4.- ROS generation in HeLa cells S5

5.- Superoxide anion generation in HeLa cells after irradiation S7

6.- Singlet oxygen measurements S8

7.- Mitochondrial potential assessment S11

8.- Autophagy induction S12

9.- Cell cycle distribution S13

10.- Apoptosis and necrosis induction S14

11.- Supplementary phototoxicity procedures S15

12.- References S17

1.- ^{1}H and ^{13}C NMR spectra and HR ESI-MS of coumarin 15

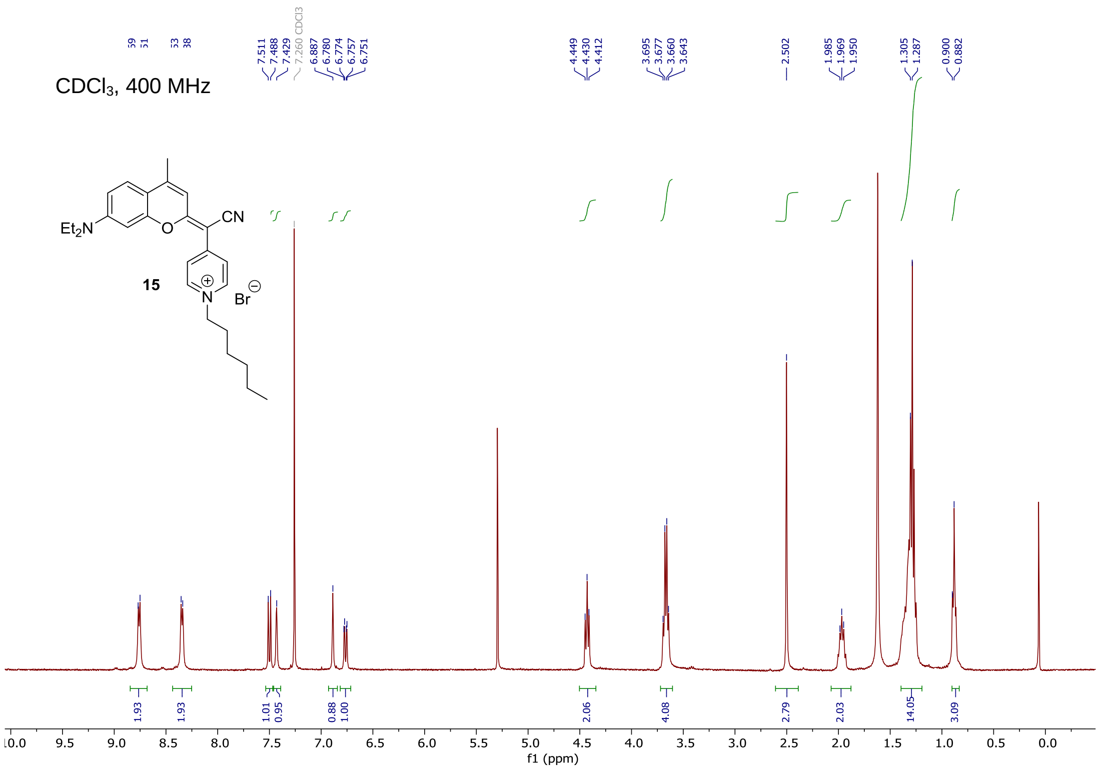

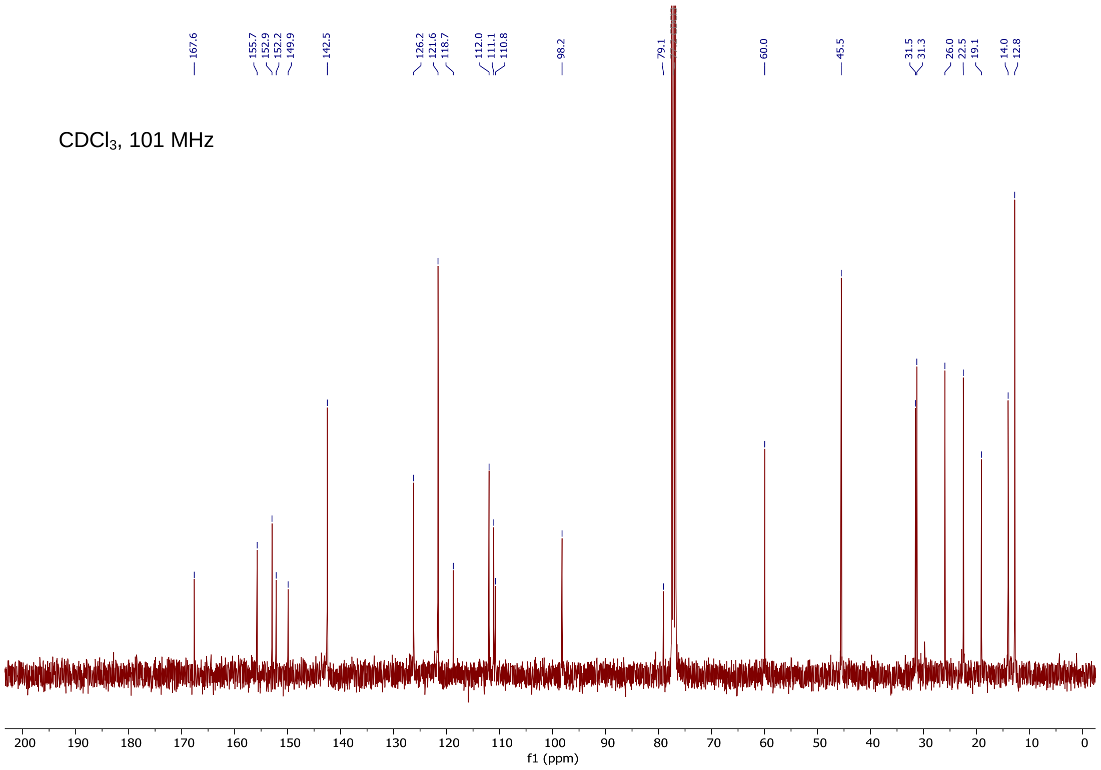

Figure S1. ^{1}H and ^{13}C NMR spectra of compound **15** in $CDCl_3$.

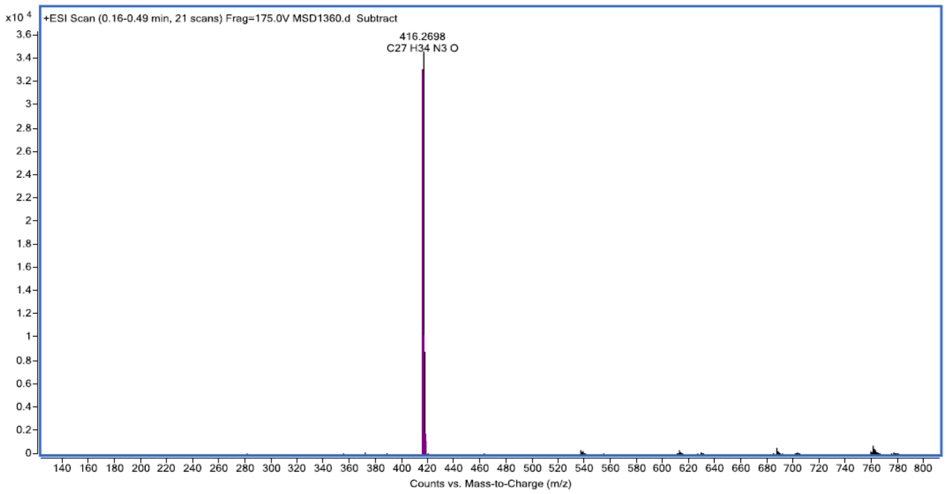

Figure S2. HR ESI-MS spectrum of coumarin **15**.

2.- Reversed-phase HPLC analysis of hit coumarins 1, 2 and 15

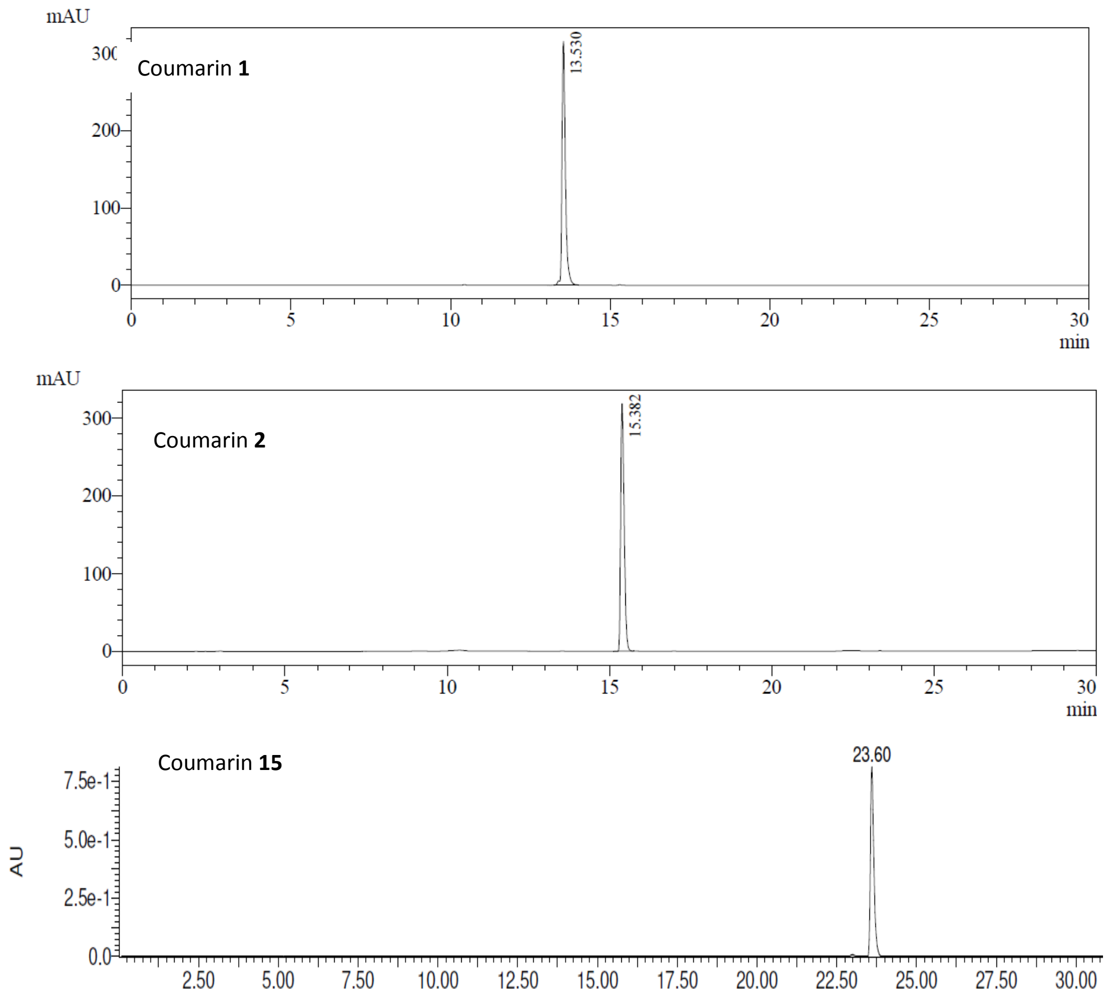

Figure S3. Reversed-phase HPLC traces of hit coumarins **1**, **2** and **15**.

3.- Fluorescence imaging

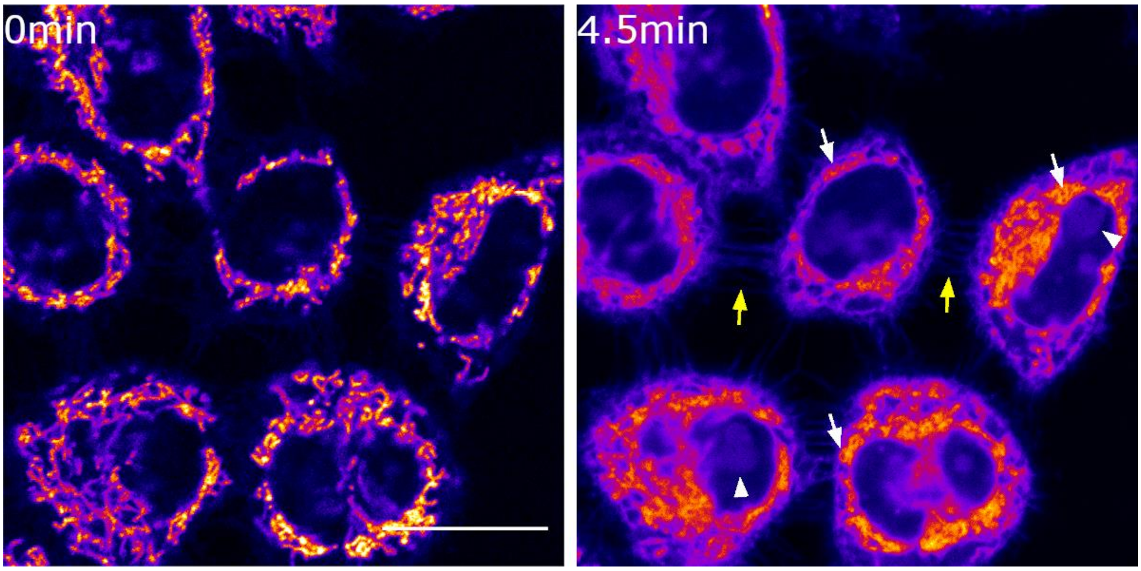

Figure S4. Cellular uptake of coumarin **15**. Single confocal planes at two different time points of image acquisition (left, t = 0; right t = 4.5 min) are shown after incubation of HeLa cells with coumarin (1.0 μM) during 30 min at 37 °C. Excitation was performed with the 561 nm laser and emission detected from 570 to 670 nm. White arrows in fluorescence images point out donut-shaped mitochondria, white arrowheads nucleoli, and yellow arrowheads filopodia staining. Scale bar: 20 μm. LUT: Fire.

Legend to Supplementary video: Time lapse movie of HeLa cells after incubation with coumarin **15** (1.0 μM) during 30 min at 37 ºC. Images were acquired every 0.58 sec. Only one focal plane was observed. Scale bar: 20 μm. LUT: Fire.

4.- ROS generation in HeLa cells

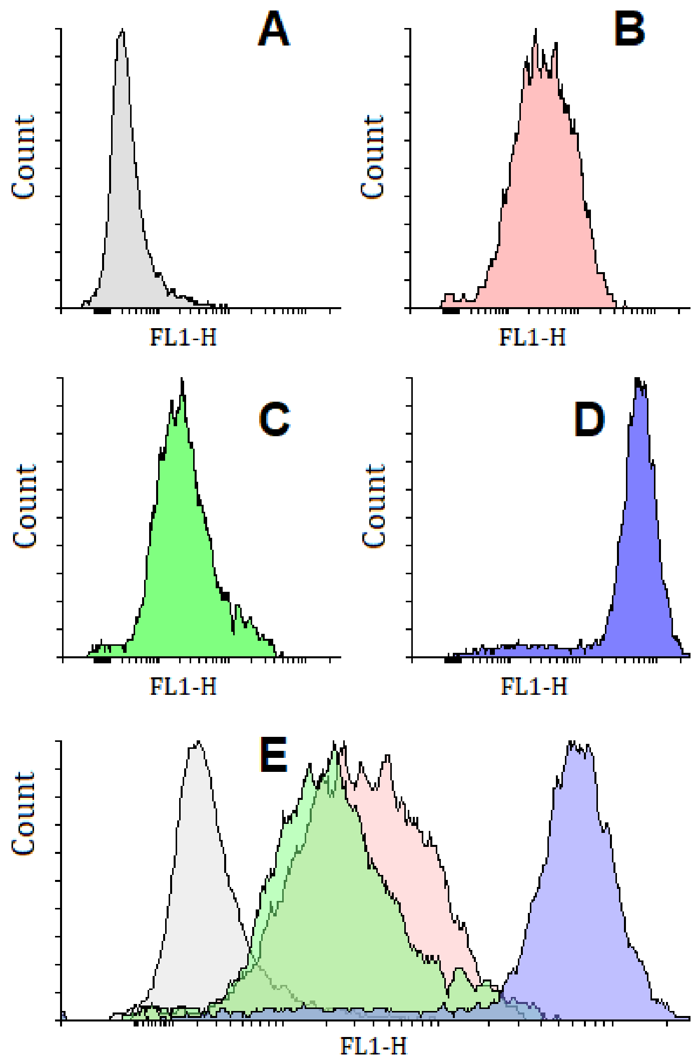

Figure S5. Representative histograms of ROS levels in HeLa cells under normoxia after light irradiation as measured by green DCF fluorescence in FL1-H channel *via* flow cytometry. A) Control cells; B) **1** (5 μM); C) **2** (5 μM); D) **15** (5 μM); E) Overlay

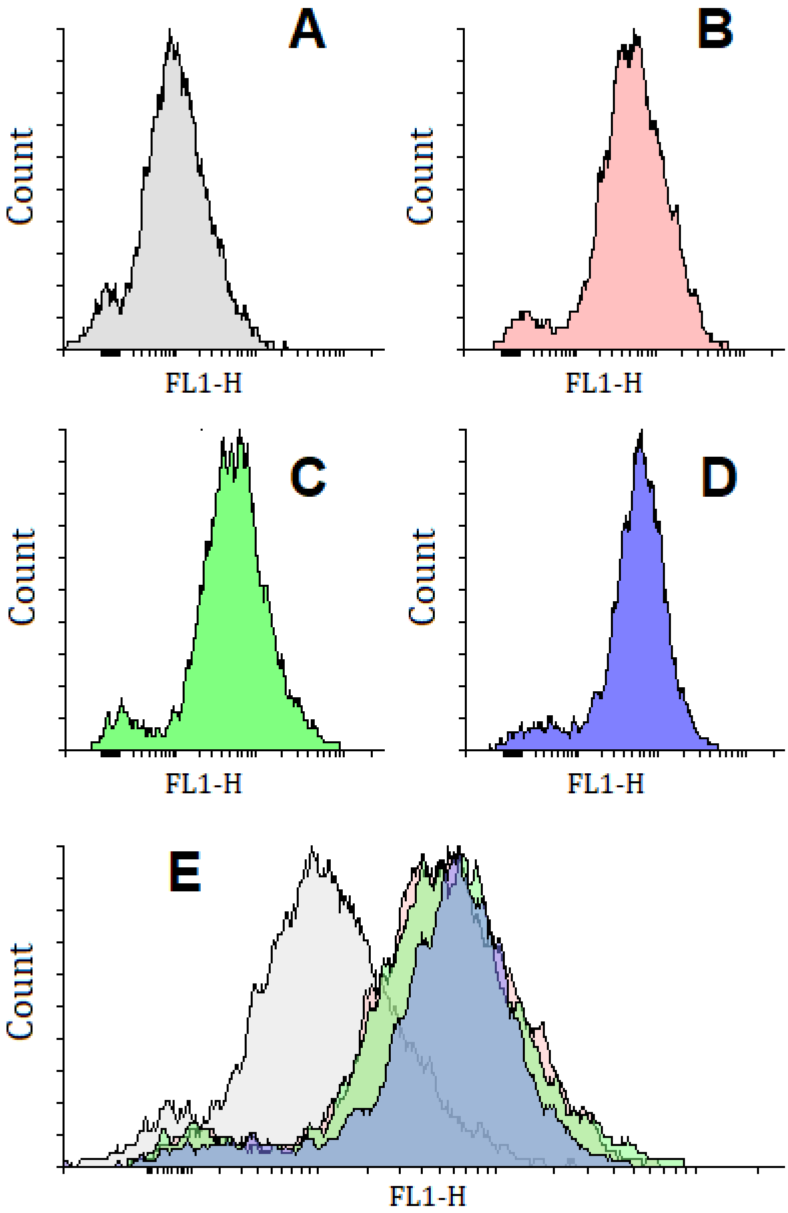

Figure S6. Representative histograms of ROS levels in HeLa cells under hypoxia after light irradiation as measured by green DCF fluorescence in FL1-H channel *via* flow cytometry. A) Control cells; B) **1** (5 µM); C) **2** (5 µM); D) **15** (5 µM); E) Overlay.

5.- Superoxide anion generation in HeLa cells after irradiation

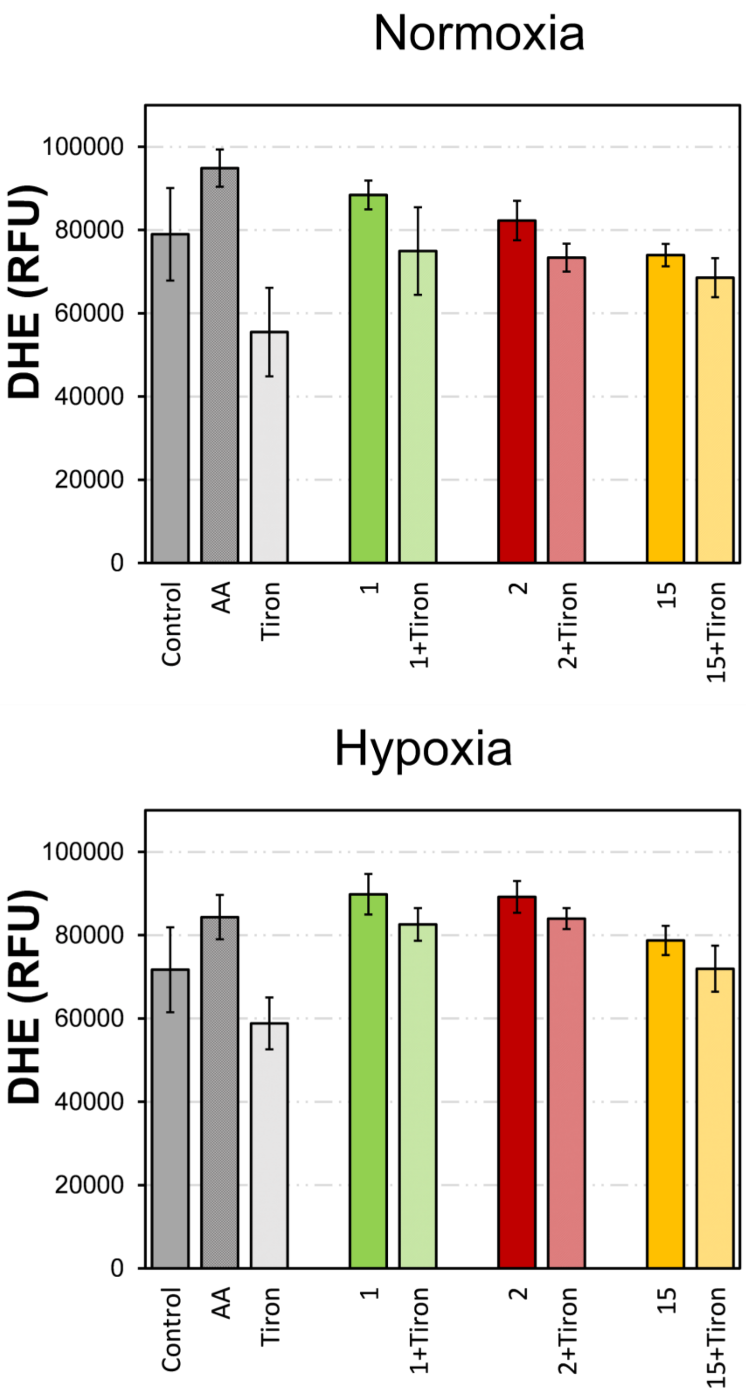

Figure S7. Dihydroethidium fluorescence (DHE) analysis for determination of superoxide anion levels after treatments with tested 5 μM compounds (1 h incubation and 1 h irradiation). Antimycin A (AA, 50 μM) used as a positive control and Tiron (5 mM) used for superoxide scavenging. Data represented as mean ± SD from two independent experiments (n=3 replicates).

6- Singlet oxygen measurements

Following previously reported procedures,[1-3] singlet oxygen quantum yields of COUPY derivatives **1**, **2** and **15** were determined in an air-saturated DCM solution (bubbled for 15 min) using 1,3-diphenylisobenzofuran (DPBF) as a chemical trap upon green light irradiation using a high-power LED source (505 nm, 100 mW cm^{-2}).[4] Upon reaction with singlet oxygen, the fluorescent scavenger DPBF decomposes into a colorless product.[5] The starting absorbance of DPBF in DCM was adjusted around 1.0 (50 µM), then COUPY derivatives were added to the cuvette and their absorbance was adjusted to 0.06 at the light irradiation wavelength (505 nm). Then, the decrease in the absorbance of DPBF at 411 nm was monitored. The linear relation of the variation in the absorbance (A_0-A_t) of DPBF at 411 nm against irradiation time was plotted (Figure S8). Singlet oxygen quantum yields where calculated by the following equation:

$$\Phi\Delta\text{s} = \Phi\Delta\text{r}\,\frac{m_s}{m_r}\,\frac{(1-10^{\text{A}\lambda\text{r}})}{(1-10^{\text{A}\lambda\text{s}})}$$

where $\Phi\Delta_r$ is the reference singlet oxygen quantum yield of methylene blue ($\Phi\Delta_r$ = 0.57 in DCM),[6] *m* are the slopes and Aλs and Aλr are the absorbance of the compounds and of the reference (methylene blue, MB) at the irradiation wavelength, respectively.
The slopes of MB and COUPY derivatives **1**, **2** and **15** were 0.21, 0.018, 0.018 and 0.044 respectively. The following singlet oxygen quantum yields were obtained: 0.049 for COUPY **1**, 0.046 for COUPY **2**, and 0.11 for COUPY **15**.

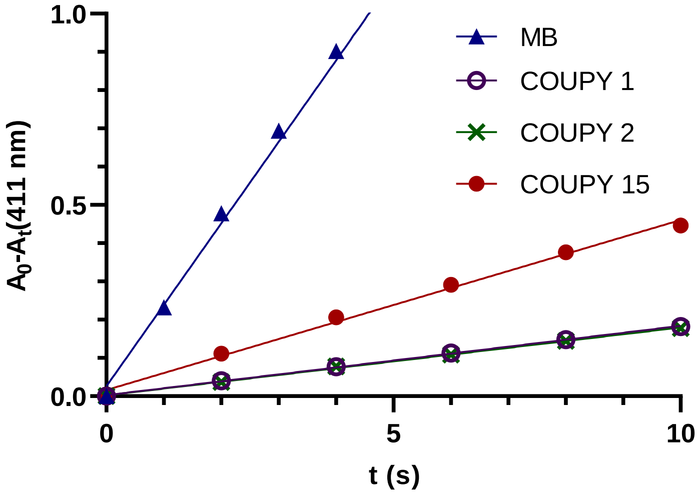

Figure S8. Plot of the changes in the absorbance (A_0-A_t) of DPBF at 411 nm against irradiation time in the presence of the standard sensitizer methylene blue and the COUPY derivatives **1**, **2** and **15** in aerated DCM.

A) DPBF

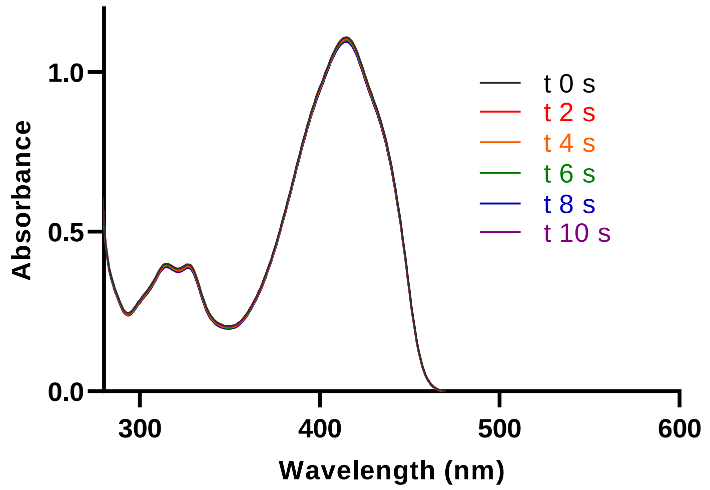

B) MB

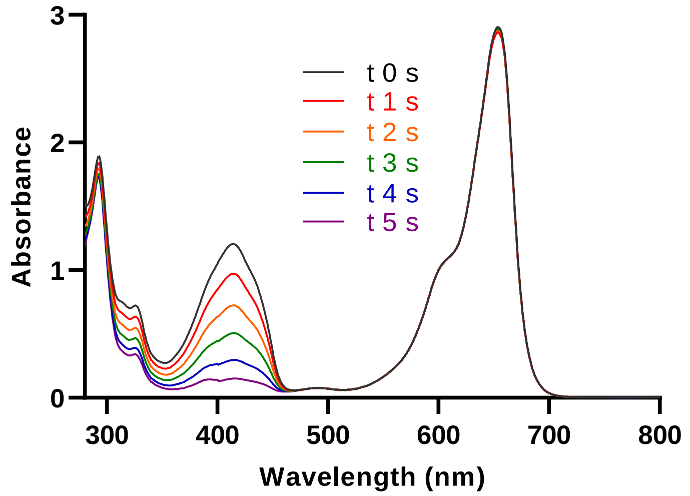

C) COUPY **1**

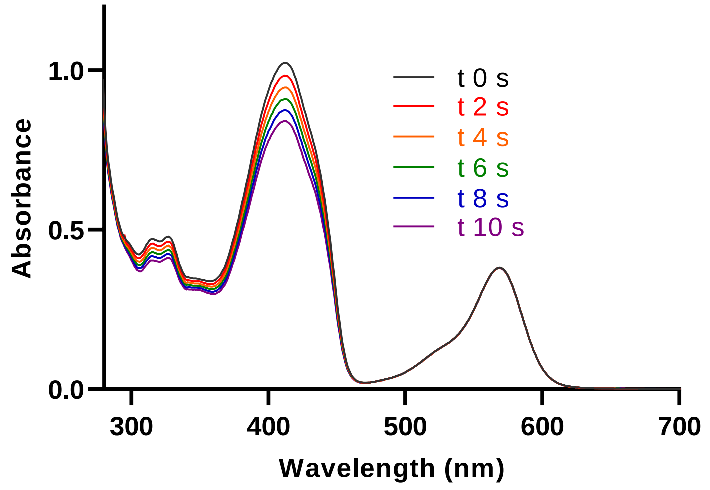

D) COUPY **2**

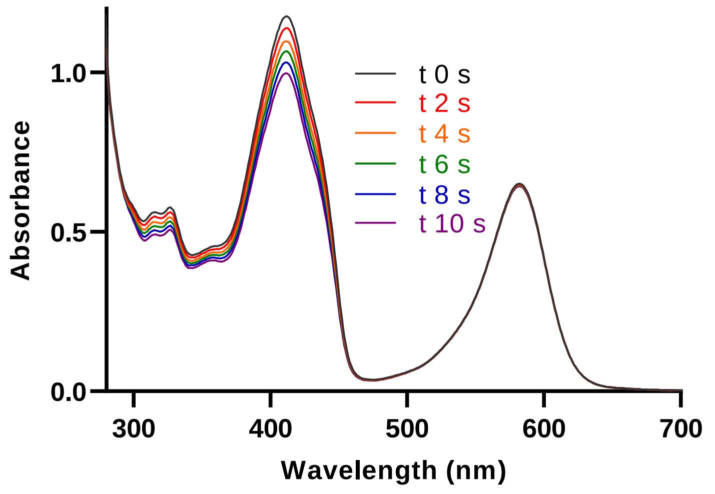

E) COUPY **15**

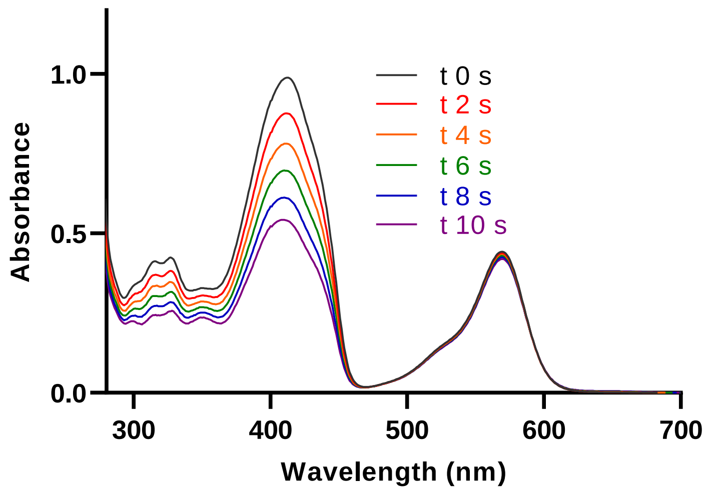

Figure S9. Changes in the absorption spectra of DPBF resulting from the irradiation with green LED light in the absence (A) and in the presence of MB (B), COUPY **1** (C), d) COUPY **2** (D) and COUPY **15** (E).

7.- Mitochondrial potential assessment

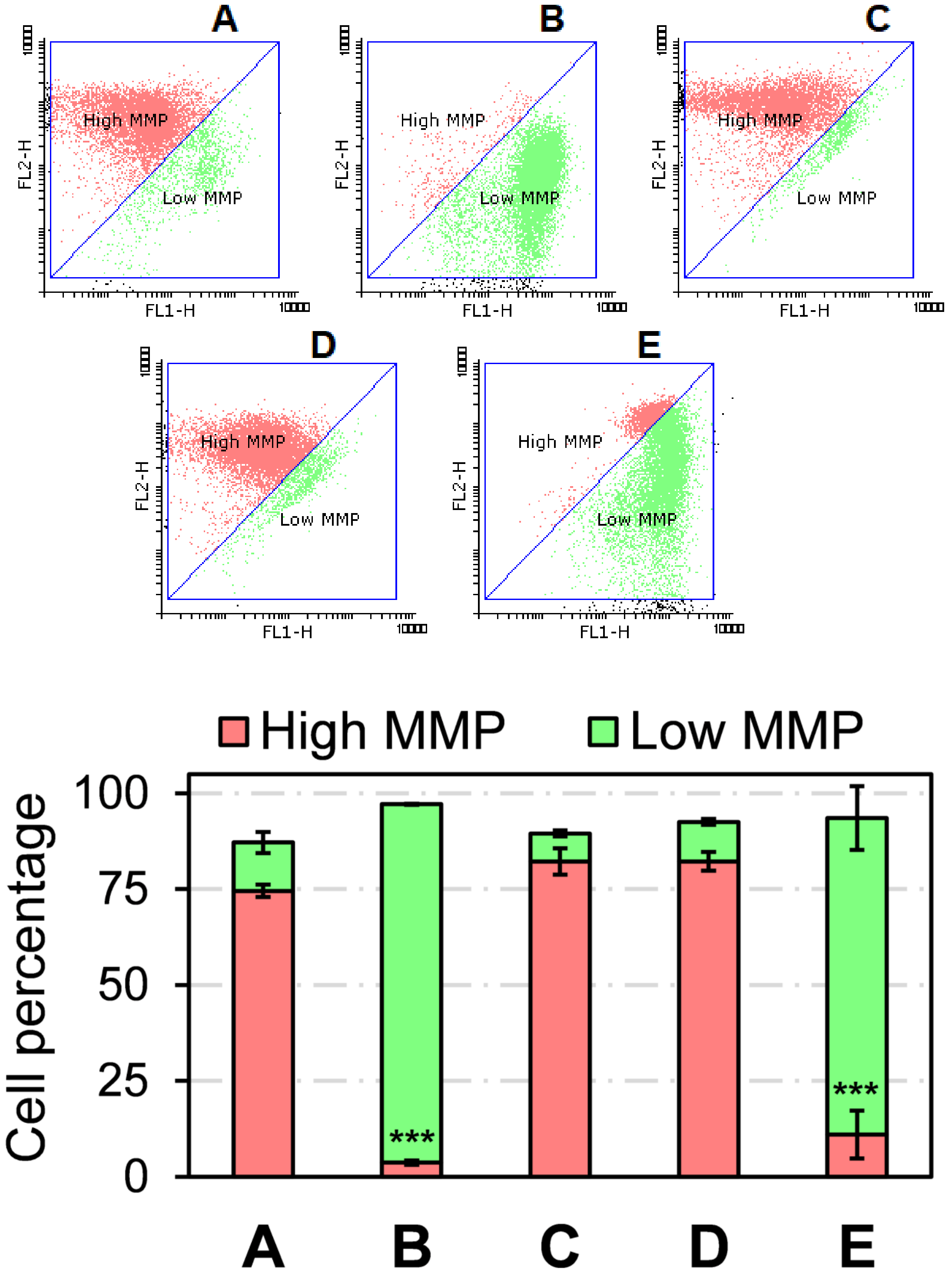

Figure S10. Flow cytometric mitochondrial membrane potential (MMP) analysis of HeLa cells after 24 h treatment with compounds. Data expressed as representative dot plots and mean ± SD from two independent experiments. A) Untreated cells; B) Antimycin A (50 µM); C) **1** (5 µM); D) **2** (5 µM); E) **15** (0.5 µM). Statistical significance between control and treatments based on *p<0.05, **p<0.01 and ***p<0.001 using unpaired t-test.

8.- Autophagy induction

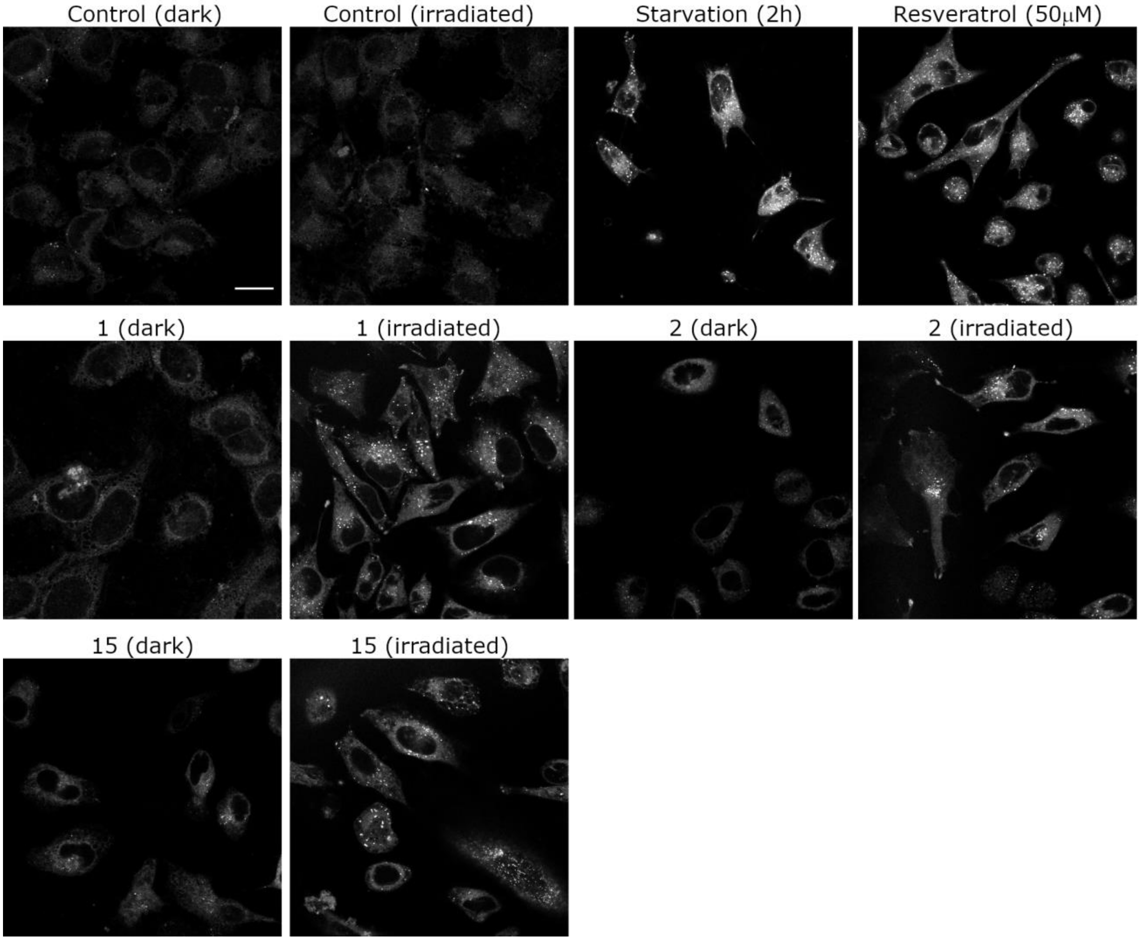

Figure S11. Confocal images of HeLa cells stained with monodansylcadaverine (0.05 mM) for 10 mins after irradiation treatments (0.5 h incubation + 1 h irradiation) with coumarins (**1** and **2**: 5 μM; **15**: 0.5 μM). Scale bar: 50 μm.

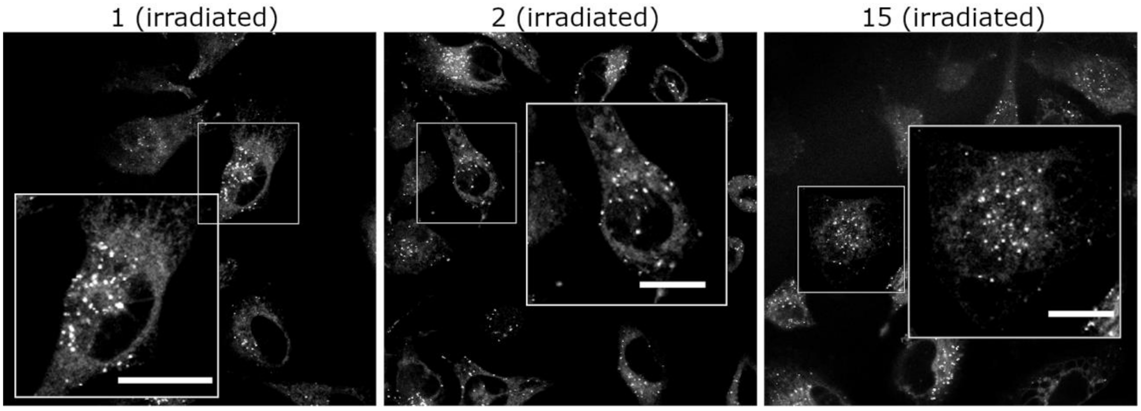

Figure S12. Detail of HeLa autophagic vesicles revealed by monodansylcadaverine staining (0.05 mM, 10 mins) after irradiation treatments (0.5 h incubation + 1 h irradiation) with coumarins (**1** and **2**: 5 μM; **15**: 0.5 μM). Scale bar: 12.5 μm.

9.- Cell cycle distribution

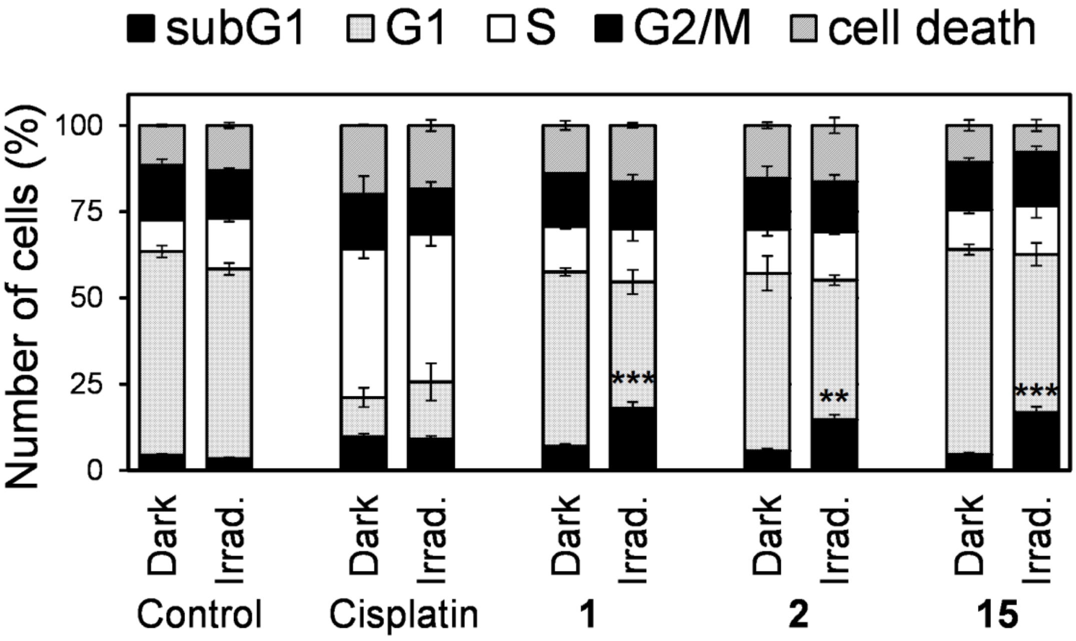

Figure S13. Cell cycle distribution analysis of HeLa cells after treatment with coumarins after irradiation or in the dark. Data represented as mean ± SD and statistical significance between dark and irradiated treatments based on *p<0.05, **p<0.01 and ***p<0.001 using unpaired t-test.

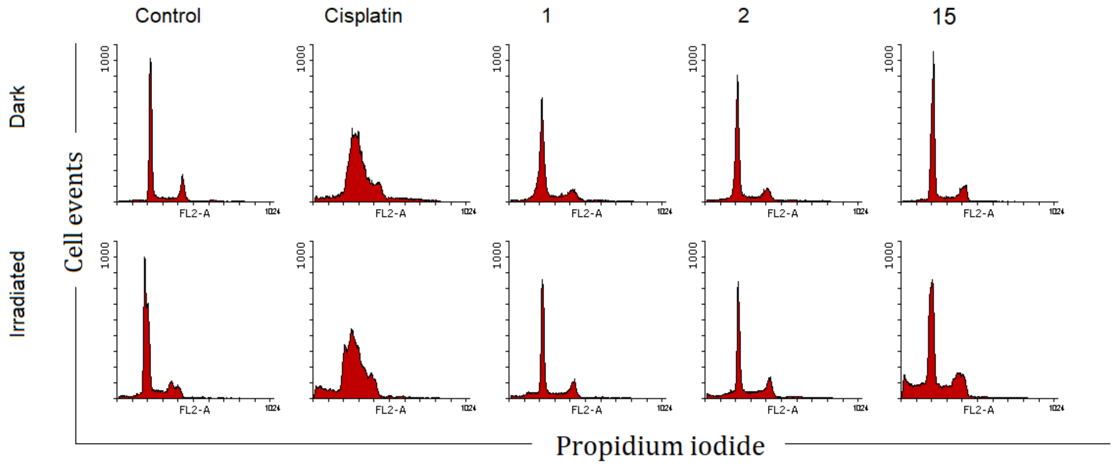

Figure S14. Representative cell cycle histograms of HeLa cells treated with coumarins in dark and after irradiation as measured by flow cytometry with propidium iodide in FL2-A channel.

10.- Apoptosis and necrosis induction

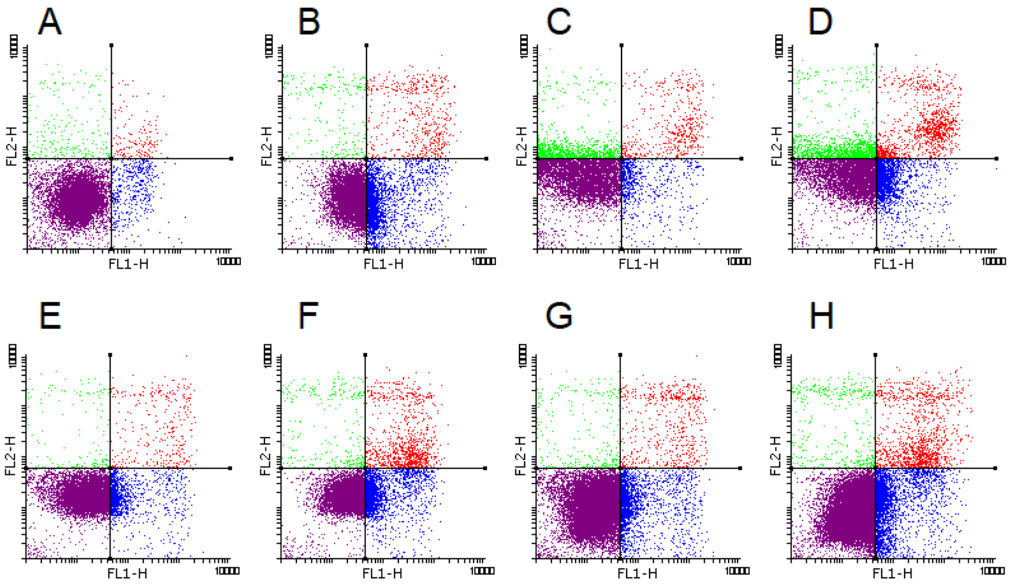

Figure S15. Representative dot plots of HeLa cells labeled with Annexin V (FL1-H) and propidium iodide (FL2-H) after treatment with coumarins in the dark or after irradiation. A) Untreated cells; B) Cisplatin (50 µM); C) **1** (5 µM); D) **1** irradiated; E) **2** (5 µM); F) **2** irradiated; G) **15** (0.5 µM); H) **15** irradiated.

11.- Supplementary phototoxicity procedures

The following phototoxicity testing procedure has been adapted and optimized from some of our previous reports.[7-10] This cell irradiation protocol was inspired by a seminal work on this topic from Bonnet *et al.*[11] Some other researchers such as Sadler *et al*.,[12] Gasser *et al*.,[13] Brabec *et al*.,[9,14] McFarland *et al.*[15,16] or Zhao *et al.*[17,18] have thus performed and developed state-of-art PDT methodologies to evaluate novel photosensitizers under both normoxia and hypoxia. In order to provide details for proper comparison to other experimental approaches, herein we describe the standard protocol used for this work.

Cells in culture were collected and dispatched in completed media into 96-well plates with 90 µL/well. Different seeding densities were tested ranging from 5.000 to 20.000 cells/well, producing similar dose-response outcomes. But lower densities (*i.e.* 5000 cells/well) were found to fit with the linear growth curve for the schedule timeline, as the photocytotoxicity experiments last up to 4 days. Seeded cells were then transferred to either normoxia or hypoxia humidified CO_2 incubators for 24 h. Hypoxia condition was set up by Tissue Culture Service at University of Murcia using nitrogen (N_2) to displace oxygen (O_2) down to a minimum of 2% in a Forma™ Steri-Cycle™ i160 incubator (ThermoFisher Scientific). Luzchem photoreactor (Canada) fitted with white lamps (final light intensity applied of 2.95 mW/cm^2 at λ_{max} =520 nm; 2.6 mW/cm^2 at λ_{max} =595 nm) was placed inside the incubator overnight. At the moment of placing the cultured plates and the photoreactor, air exposure to hypoxia incubator rises the O_2 concentration. However, it just takes some minutes (from 5 to 35 min approximately) until the O_2 concentration asymptotically approximates the desired value (2 %).

The following day, serial dilutions of the compounds were prepared in sterile 96-well plates in cell media at the biosafety cabinet. Initial dilutions were prepared in serial across 8 concentrations that are 250X concentrated using DMSO as a solvent. Then, cell media was used to further dilute the samples until 10X. These dilutions will give final concentrations in the range of 0 to 100 µM by dispensing 10 µL/well into the cell plates (0.4% DMSO v/v). When dilutions are ready, cell plates can be taken out of the incubators to apply treatments. In the case of hypoxia incubator, the use of segmented inner doors of the incubators is strongly recommended since it will decrease gas consumption and minimize air exposure.[19] Also, less nitrogen is needed to recover the inner O_2 concentration after opening and closing the small, segmented doors of the incubator. Serial dilution treatments were then applied to every plate. At this point, all sample and control wells have 100 µL/well; control wells receiving 10 µL of complete media.

Cell culture plates were then transferred to the incubator for 1 h incubation in the dark. All the plates subjected to light irradiation included untreated controls to verify that cell viability was not affected. After 1 h incubation in the dark, photoreactor was switched on for 1 h light treatment. Non-irradiated plates were placed under dark conditions inside the incubator (and wrapped with aluminum paper) directly for 2 h in the dark. After treatments, photoreactor was switched off, removed from the incubator and the plates taken out to remove drug-containing media. Fresh complete media was added to every treated well and a cell recovery period of 48 h was allowed. In the case of hypoxic incubators, again air exposure may rise O_2 concentration due to doors opening and closing, but stabilization will rapidly occur within the next minutes.

After recovery period, medium was aspirated by suction, cells were loaded with 50 µL of MTT solution (1 mg/mL) for additional 4 h, then removed and 50 µL DMSO was added to solubilize the purple formazan crystals formed in active cells. The absorbance was measured at 570 nm using a microplate reader (FLUOstar Omega) and the IC_{50} values were calculated based on the inhibitory rate curves using the following equation:

$$I = \frac{I_{max}}{1 + \left(\frac{IC_{50}}{C}\right)^{n}}$$

Where I represent the percentage inhibition of viability observed, I_{max} is the maximal inhibitory effect, IC_{50} is the concentration that inhibits 50% of maximal growth, C is the concentration of the treatment and n is the slope of the semi-logarithmic dose-response sigmoidal curves. The non-linear fitting was performed using SigmaPlot 14.0 software. All experiments were performed at least in two independent studies with n=3 replicates per concentration level.

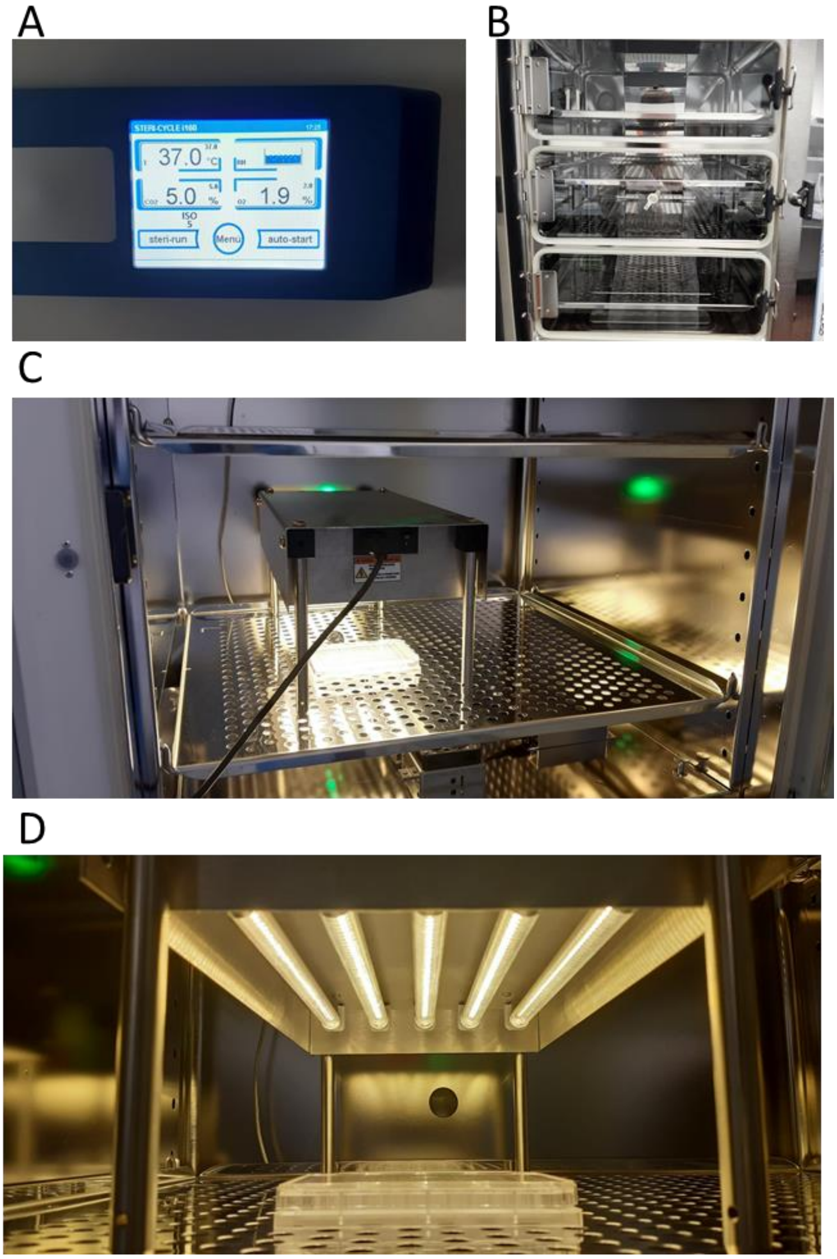

Figure S16. Experimental set up for cell plate irradiation protocol. A) Forma™ Steri-Cycle™ i160 incubator settings for hypoxic condition. B) Whole incubator view with segmented inner doors used to minimize air exposure. C) Luzchem photoreactor set up for plate irradiation. D) Detailed view of photoreactor irradiation with visible light.

12.- References

(1) Adarsh, N.; Avirah, R. R.; Ramaiah, D. Tuning photosensitized singlet oxygen generation efficiency of novel aza-BODIPY dyes. *Org. Lett*. **2010**, *12* (24), 5720-5723. https://doi.org/10.1021/ol102562k.

(2) Li, W.; Li, L.; Xiao, H.; Qi, R.; Huang, Y.; Xie, Z.; Jing, X.; Zhang, H. Iodo-BODIPY: a visible-light-driven, highly efficient and photostable metal-free organic photocatalyst. *RSC Adv*. **2013**, *3*, 13417-13421. https://doi.org/10.1039/c3ra40932e.

(3) Lv, Z.; Wei, H.; Li, Q.; Su, X.; Liu, S.; Zhang, K. Y.; Lv, W.; Zhao, Q.; Li, X.; Huang, W. Achieving efficient photodynamic therapy underboth normoxia and hypoxia using cyclometalatedRu(II) photosensitizer through type I photochemicalprocess. *Chem. Sci*. **2018**, *9*, 502-512. https://doi.org/10.1039/c7sc03765a.

(4) López-Corrales, M.; Rovira, A.; Gandioso, A.; Bosch, M.; Nonell, S.; Marchán, V. Transformation of COUPY Fluorophores into a Novel Class of Visible-Light-Cleavable Photolabile Protecting Groups. *Chem. Eur. J.* **2020**, *26* (69), 16222-16227. https://doi.org/10.1002/chem.202002314.

(5) Zhang, X.; Zhang, G. Q.; Zhu, J. Methylated unsymmetric BODIPY compounds: synthesis, high fluorescence quantum yield and long fluorescence time. *J. Fluoresc*. **2019**, *29* (2), 407-416. https://doi.org/10.1007/s10895-019-02349-5.

(6) Yoshiharu, U. Determination of quantum yield of singlet oxygen formation by photosensitization. *Chem. Lett*. **1973**, *2* (7), 743-744. https://doi.org/10.1246/cl.1973.743.

(7) Ballester, F. J.; Ortega, E.; Bautista, D.; Santana, M. D.; Ruiz, J. Ru(II) Photosensitizers Competent for Hypoxic Cancers via Green Light Activation. *Chem. Commun.* **2020**, *56* (71), 10301–10304. https://doi.org/10.1039/D0CC02417A.

(8) Ortega, E.; Pérez-Arnaiz, C.; Rodríguez, V.; Janiak, C.; Busto, N.; García, B.; Ruiz, J. A 2-(Benzothiazol-2-Yl)-Phenolato Platinum(II) Complex as Potential Photosensitizer for Combating Bacterial Infections in Lung Cancer Chemotherapy†. *Eur. J. Med. Chem.* **2021**, *222*, 113600. https://doi.org/10.1016/j.ejmech.2021.113600.

(9) Novohradsky, V.; Rovira, A.; Hally, C.; Galindo, A.; Vigueras, G.; Gandioso, A.; Svitelova, M.; Bresolí-Obach, R.; Kostrhunova, H.; Markova, L.; Kasparkova, J.; Nonell, S.; Ruiz, J.; Brabec, V.; Marchán, V. Towards Novel Photodynamic Anticancer Agents Generating Superoxide Anion Radicals: A Cyclometalated IrIII Complex Conjugated to a Far-Red Emitting Coumarin. *Angew. Chem. Int. Ed. Engl.* **2019**, *58* (19), 6311–6315. https://doi.org/10.1002/anie.201901268.

(10) Novohradsky, V.; Vigueras, G.; Pracharova, J.; Cutillas, N.; Janiak, C.; Kostrhunova, H.; Brabec, V.; Ruiz, J.; Kasparkova, J. Molecular Superoxide Radical Photogeneration in Cancer Cells by Dipyridophenazine Iridium(III) Complexes. *Inorg. Chem. Front.* **2019**, *6* (9), 2500–2513. https://doi.org/10.1039/C9QI00811J.

(11) Hopkins, S. L.; Siewert, B.; Askes, S. H. C.; Veldhuizen, P.; Zwier, R.; Heger, M.; Bonnet, S. An in Vitro Cell Irradiation Protocol for Testing Photopharmaceuticals and the Effect of Blue, Green, and Red Light on Human Cancer Cell Lines. *Photochem. Photobiol. Sci.* **2016**, *15* (5), 644–653. https://doi.org/10.1039/C5PP00424A.

(12) Huang, H.; Banerjee, S.; Qiu, K.; Zhang, P.; Blacque, O.; Malcomson, T.; Paterson, M. J.; Clarkson, G. J.; Staniforth, M.; Stavros, V. G.; Gasser, G.; Chao, H.; Sadler, P. J. Targeted Photoredox Catalysis in Cancer Cells. *Nat. Chem.* **2019**, *11* (11), 1041–1048. https://doi.org/10.1038/s41557-019-0328-4.

(13) Karges, J.; Kuang, S.; Maschietto, F.; Blacque, O.; Ciofini, I.; Chao, H.; Gasser, G. Rationally Designed Ruthenium Complexes for 1- and 2-Photon Photodynamic Therapy. *Nature Commun.* **2020**, *11* (1), 3262. https://doi.org/10.1038/s41467-020-16993-0.

(14) Novohradsky, V.; Markova, L.; Kostrhunova, H.; Kasparkova, J.; Ruiz, J.; Marchán, V.; Brabec, V. A Cyclometalated IrIII Complex Conjugated to a Coumarin Derivative Is a Potent

Photodynamic Agent against Prostate Differentiated and Tumorigenic Cancer Stem Cells. *Chem. Eur. J.* **2021**, *27* (33), 8547–8556. https://doi.org/10.1002/chem.202100568.

(15) Roque, J. A.; Barrett, P. C.; Cole, H. D.; Lifshits, L. M.; Shi, G.; Monro, S.; Dohlen, D. von; Kim, S.; Russo, N.; Deep, G.; Cameron, C. G.; Alberto, M. E.; McFarland, S. A. Breaking the Barrier: An Osmium Photosensitizer with Unprecedented Hypoxic Phototoxicity for Real World Photodynamic Therapy. *Chem. Sci.* **2020**, *11* (36), 9784–9806. https://doi.org/10.1039/D0SC03008B.

(16) McFarland, S. A.; Mandel, A.; Dumoulin-White, R.; Gasser, G. Metal-Based Photosensitizers for Photodynamic Therapy: The Future of Multimodal Oncology? *Curr. Opin. Chem. Biol.* **2019**, *56*, 23–27. https://doi.org/10.1016/j.cbpa.2019.10.004.

(17) Liu, X.; Li, G.; Xie, M.; Guo, S.; Zhao, W.; Li, F.; Liu, S.; Zhao, Q. Rational Design of Type I Photosensitizers Based on Ru(II) Complexes for Effective Photodynamic Therapy under Hypoxia. *Dalton Trans.* **2020**, *49* (32), 11192–11200. https://doi.org/10.1039/D0DT01684E.

(18) Lv, Z.; Wei, H.; Li, Q.; Su, X.; Liu, S.; Zhang, K. Y.; Lv, W.; Zhao, Q.; Li, X.; Huang, W. Achieving Efficient Photodynamic Therapy under Both Normoxia and Hypoxia Using Cyclometalated Ru(II) Photosensitizer through Type I Photochemical Process. *Chem. Sci.* **2018**, *9* (2), 502–512. https://doi.org/10.1039/C7SC03765A.

(19) Wenger, R. H.; Kurtcuoglu, V.; Scholz, C. C.; Marti, H. H.; Hoogewijs, D. Frequently Asked Questions in Hypoxia Research. *Hypoxia (Auckl)* **2015**, *3*, 35–43. https://doi.org/10.2147/HP.S92198.
